# Treatment of diabetic ulcers with Chuang Ling Ye: systems pharmacology and functional validation reveal the mechanism of angiogenesis and inflammation abatement driven by the PI3K-AKT signaling pathway

**DOI:** 10.3389/fphar.2025.1644541

**Published:** 2025-10-27

**Authors:** Xiao Feng, Nan Yi, Ya Zhao, Cunyu Zhang, Yue Chen, Qian Wang, Xindong Yin, Yuanzi Zhou, Ying Liu, Chao Pan, Gaoyuan Wang, Chaoqun Ma

**Affiliations:** ^1^ Department of General Surgery, Jiangsu Province Hospital of Chinese Medicine, Affiliated Hospital of Nanjing University of Chinese Medicine, Nanjing, China; ^2^ The First Clinical Medical College, Nanjing University of Chinese Medicine, Nanjing, China

**Keywords:** diabetic ulcers, PI3K-Akt pathway, angiogenesis, inflammation, macrophage polarization

## Abstract

**Aim of the study:**

Diabetic ulcer (DU), a major complication of diabetes, is characterized by complex pathophysiology and limited therapeutic options. Chuang Ling Ye (CLY) has demonstrated significant therapeutic efficacy in the treatment of DU. This study investigates the therapeutic potential of CLY for DU *in vivo* and *in vitro*, examining its mechanisms at tissue, cellular, and molecular levels.

**Methods:**

A UHPLC-MS/MS methodology was employed to identify metabolites in CLY. Network pharmacology was utilized to construct a metabolite-target network, and GO and KEGG enrichment analyses were performed to predict potential biological mechanisms of CLY in DU treatment. Furthermore, the therapeutic efficacy of CLY against DU was validated through *in vitro* and *in vivo* experiments. The mechanism of action of CLY in DU treatment was further verified using Western blot and flow cytometry.

**Results:**

A total of 357 metabolites were identified in CLY using UHPLC-MS/MS. Our data indicate that CLY significantly promoted DU healing *in vivo*. *In vitro* experiments revealed that CLY ameliorated high glucose-induced dysfunction in HUVEC cells and upregulated the expression of angiogenesis-related proteins VEGF and CD31. Additionally, CLY significantly reduced the release of inflammatory factors in lipopolysaccharide-induced THP-1 cells, confirming its anti-inflammatory properties. These effects were attenuated following inhibition of the PI3K-Akt signaling pathway with LY294002. Consistent results were observed *in vivo*, elucidating the therapeutic efficacy and mechanistic basis of CLY in DU treatment.

**Conclusion:**

CLY promotes DU healing by attenuating inflammatory responses and enhancing angiogenesis through activation of the PI3K-Akt signaling pathway.

## 1 Introduction

Diabetes mellitus (DM) is a common disease with various complications due to disease progression or poor glycemic control, with diabetic ulcers (DUs) being one of the most common complications (Armstrong et al., 2023). DUs are characterized by persistent inflammation, impaired angiogenesis, and chronic oxidative stress, all of which contribute to delayed wound healing and a heightened risk of recurrence (Deng et al., 2023; Voza et al., 2024). Epidemiological evidence suggests that the lifetime risk of developing DUs in individuals with diabetes may reach 34%, with recurrence rates further exacerbating long-term clinical outcomes (McDermott et al., 2023). Owing to the complex pathophysiology of DUs, severe cases may progress to limb necrosis, necessitating amputation. This outcome imposes substantial psychological burdens on patients and places significant strain on public healthcare systems. Current clinical interventions, including skin grafts, growth factor therapies, and advanced biomaterials, are frequently limited by high costs, inconsistent efficacy, or adverse effects, thereby compromising their utility. Consequently, there is an urgent need to develop cost-effective, efficient, and safer therapeutic strategies for DUs managements.

Chuang Ling Ye (CLY) (Approval No. Z04000525), a topical botanical drug formulation developed by Jiangsu Provincial Hospital of Traditional Chinese Medicine, has been clinically utilized for three decades and widely applied to diverse wound types. Its composition includes Rheum palmatumL. [Polygonaceae; Rhei radix et rhizoma], Terminalia chebulaRetz. [Combretaceae; Chebulae fructus], Carthamus tinctoriusL. [Asteraceae; Carthami flos], and Abelmoschus manihot (L.) Medik. [Malvaceae; Abelmoschi corolla]. Botanical nomenclature was verified using the Medicinal Plant Names Services (MPNS, http://mpns.kew.org) and the Chinese Pharmacopoeia (https://ydz.chp.org.cn). A randomized controlled trial demonstrated CLY’s potent anti-inflammatory properties in idiopathic granulomatous mastitis treatment, significantly lowering serum levels of IL-1β, IFN-γ, and TNF-α in patients (Xue et al., 2020). Although CLY is a commonly used treatment for DU in clinical settings, the efficacy and underlying mechanisms of its action remains unknow and requires further investigation.

**FIGURE 1:**
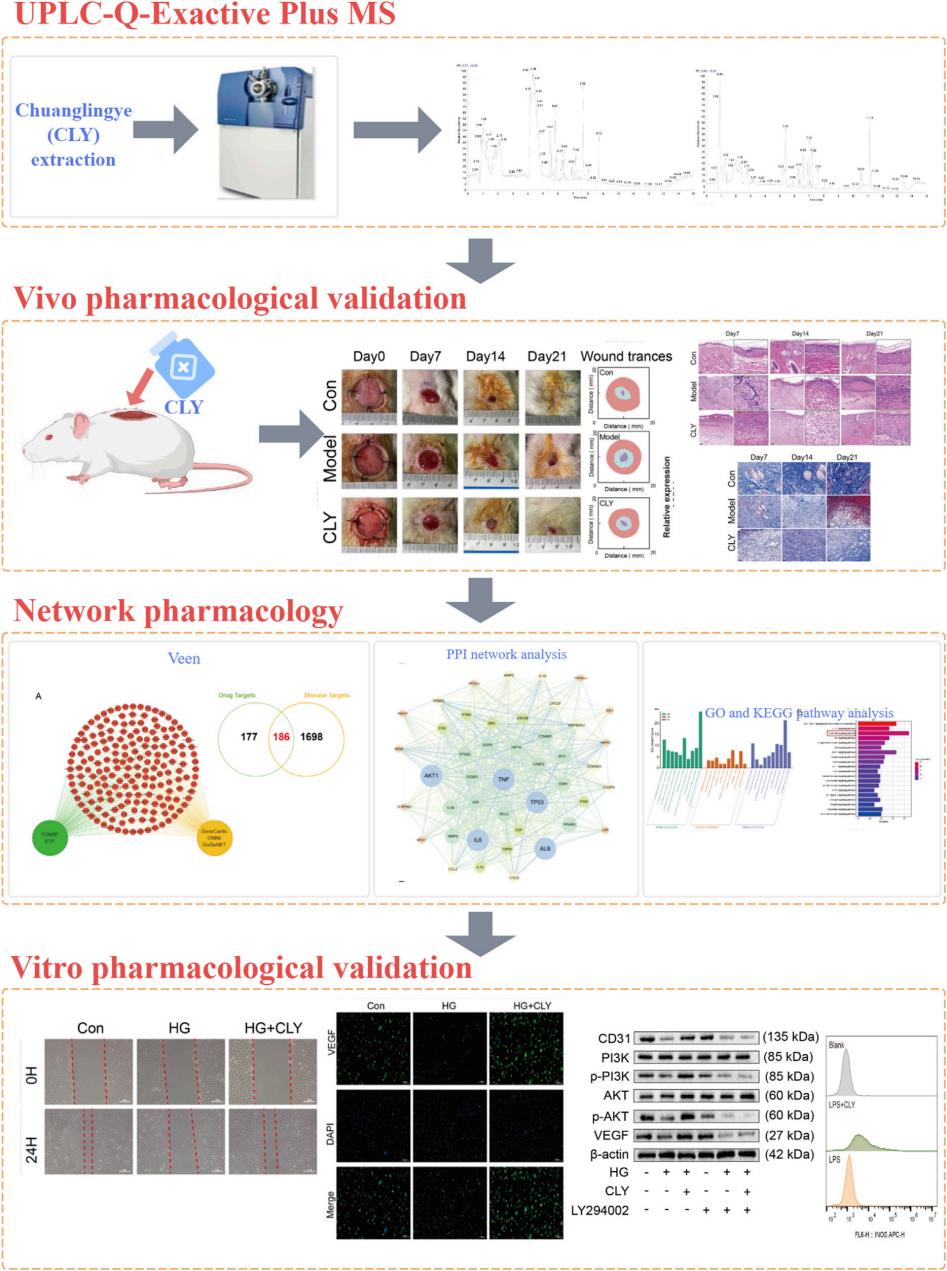
Graphical abstract.

Network pharmacology is a new research tool in modern Chinese medicine research. It provides a modern medical explanation for the therapeutic mechanisms of traditional medicines (Hao and Xiao, 2014). Network pharmacology explores the effect of an active metabolite on a target through target-to-target interactions and visualises them using a network. In this study, we employed UHPLC-MS/MS combined with network pharmacology to investigate the interactions between CLY’s bioactive metabolites and their molecular targets, followed by integrated *in vivo* and *in vitro* experiments to elucidate its therapeutic mechanisms in DU treatment. The graphical abstract of the experimental workflow is shown in Figure 1.

## 2 Methods

### 2.1 Preparation of CLY

CLY was prepared by the Pharmaceutical Factory of Jiangsu Provincial Hospital of Traditional Chinese Medicine (Approval No.: JSZBZ20090873Z). Its composition is presented in Table 1. All crude drugs were authenticated by licensed hospital herbalists to ensure pharmacopeial quality, and the preparation strictly adheres to the ConPhyMP Guidelines for standardized methodology in botanical preparations [7]. Each botanical drug was soaked in 10 times its volume of water for 30 min, boiled over high heat, and then simmered for 40 min. The decoction was filtered through gauze, and the residue was reboiled with 8 times its volume of water for 1 h. The combined filtrates were concentrated to 250 mL of fluid extract.

**TABLE 1:** The composition of Chuanglingye (CLY).

Chinese name	Latin name	Source species	Parts used	Weight (g)
Dahuang	Rhei Radix et Rhizoma	Rheum palmatum L.	Root	50
Hezi	Chebulae Fructus	Terminalia chebula Retz.	Fruit	25
Honghua	Carthami Flos	Carthamus tinctorius L.	Flower	25
Huangshukuihua	Abelmoschi Corolla	Abelmoschus manihot (L.) Medicus	Flower	12.5

CLY (250 mL) was evaporated using a 70 °C water bath to concentrate the solution. Following filtration, the concentrated extract was dried and pulverized into a powder. The resulting powder (20.8 g) was stored at 4 °C for subsequent use in cellular experiments.

### 2.2 UHPLC-MS/MS analysis

Chromatographic separation and mass spectrometric characterization of CLY constituents were achieved using an integrated ultra-high-performance liquid chromatography-tandem mass spectrometry (UHPLC-MS/MS) platform. The system incorporated a Shimadzu LC-30 UHPLC unit coupled to a Thermo Scientific Q Exactive™ Plus hybrid quadrupole-Orbitrap mass spectrometer, employing an ACQUITY UPLC^®^ HSS T3 reversed-phase column (2.1 × 100 mm, 1.8 μm; Waters) maintained at 40 °C within a thermostated column compartment. Sample injections of 4 μL were delivered at a constant flow rate of 0.3 mL/min using a binary mobile phase comprising solvent A (aqueous 0.1% v/v formic acid) and solvent B (neat acetonitrile). The gradient profile progressed as follows: initial isocratic conditions (0–2 min, 0% B); linear gradient to 48% B (2–6 min); further linear transition to 100% B (6–10 min); isocratic wash at 100% B (10–12 min); step gradient to initial conditions (12–12.1 min, 100→0% B); and column re-equilibration (12.1–15 min, 0% B). Dual-polarity electrospray ionization was implemented with optimized parameters: ion spray voltages of +3.8 kV (positive mode) and −3.2 kV (negative mode); sheath and auxiliary gas flows at 30 and 5 arbitrary units respectively; capillary temperature stabilized at 350 °C; S-lens RF level set to 50. Post-acquisition data processing via MS-DIAL software enabled automated peak detection, retention time alignment, and quantitative area integration. Structural annotations were verified through comparative analysis against authenticated standards and spectral libraries by Shanghai BIOPROFILE Biotechnology Co., Ltd., ensuring rigorous compound identification.

### 2.3 Establishment of diabetic rat model

Specific pathogen free (SPF) grade, 8 weeks old, male Sprague-Dawley (SD) rats were used to establish DU model. The experimental animals were purchased from Sparfo (Suzhou) Biotechnology Co., Ltd. with license number SCXK (Su) 2022–0006, and all animal experiments were approved by the Animal Ethics Committee of the Affiliated Hospital of Nanjing University of Traditional Chinese Medicine with approval number 2023DW-010-01. During the study period, the animals were housed in conditions of constant temperature (25 °C ± 2 °C), constant humidity (50%–60%), and diurnal light (12 h light/12 h dark), during which free access to water and food was allowed. After 1 week of adaptation, the rats were randomly divided into NC, DM and CLY groups. Animals were allocated to three experimental groups (n = 6 per group). Except for the NC group, all rats were fed with high-fat chow for 4 weeks. After 4 weeks, all rats were fasted for 12 h and their basal blood glucose levels were determined using the tail-prick blood method and a glucometer. The DM and CLY groups received an intraperitoneal injection of 40 mg/kg of streptozotocin (STZ), whereas the rats in the NC group received an intraperitoneal injection of an equal volume of citrate-sodium citrate buffer (PH4.2). During the next 2 weeks, we monitored the random blood glucose levels of the rats by performing rat tail-prick blood measurements every 2 days. When the random blood glucose value of the rats reached 16.7 mmol/L, it was determined that the rat DM model was successfully established.

### 2.4 Construction and treatment of diabetic wounds in rats

On the day of modelling, rats were weighed and anaesthetised by intraperitoneal injection of 1% sodium pentobarbital at a body weight ratio of 40 mg/kg. The backs of the rats were clipped and prepared for skin treatment using a lancet. Along the midline of the dorsal spine, a circular mark with a diameter of 16 mm and an area of 2.00 cm^2^ was made, and the entire skin and subcutaneous connective tissue within the marked area were excised and bluntly detached until they reached the deep fascial layer. A plastic ring (inner diameter 16 mm, outer diameter 20 mm) was fixed with 4 sutures to the wound margin and haemostasis was performed with sterile gauze. According to the TIME principle, saline and povidone-iodine were used for dressing changes in the NC and diabetic control (untreated) group, and povidone-iodine and CLY were used for dressing changes in the CLY group. CLY (450 mg/mL), was saturated onto sterile gauze, which was then cut to an appropriate size for the wound using sterile scissors. The gauze was applied to cover the center of the wound, followed by bandaging with additional gauze. The dressing was changed once daily. The diabetic model group received identical wound care, excluding CLY, ensuring that the presence of CLY was the only experimental difference.

### 2.5 Hematoxylin and eosin (HE) staining and masson staining

The isolated rat trauma tissue was placed in 4% paraformaldehyde for 1 week. Afterwards, the tissues were carefully prevented from breaking at the bottom of the embedding container. Subsequently, dehydration, waxing and embedding were performed according to standard protocols. Upon completion, the tissue was sectioned and placed on slides for spreading and baking steps. For further observation of pathological changes within the traumatised tissue, the sections were dewaxed and dehydrated using xylene and ethanol and stained using hematoxylin and eosin staining as well as Masson staining techniques, and the stained sections were observed under a light microscope and analysed.

### 2.6 Cell culture

HUVEC and THP-1 cells were purchased from SUNNCELL Technology Company (Wuhan, China), both inoculated in specialised medium (HUVEC: GZ12103, Servicebio, China; THP-1: GZ10907, Servicebio, China) and cultured in a 37 °C, 5% CO2 cell culture incubator for static culture, observe the cell growth status, and replace the cell culture on time. For hyperglycemic stimulation, HUVEC cells were exposed to high glucose (25 mM D-glucose) for 48 h. LY294002 was dissolved in dimethyl sulfoxide (DMSO), and an independent vehicle control group was included. No detectable effects from the solvent itself were observed, confirming that the reported effects were solely attributable to the inhibitor.

### 2.7 Western blotting

Protein samples were separated by SDS-PAGE and transferred to methanol-activated PVDF membranes. Subsequently, the PVDF membrane was placed in TBST solution containing 5% skimmed milk powder for 1 h for closure. The PVDF membranes at the end of the closure were incubated together with antibodies containing VEGF, CD31, PI3K, p-PI3K, AKT, p-AKT and β-actin, respectively, at 4 °C overnight. At the end of the incubation, the membrane was washed three times with TBST for 5 min each time. After washing, the secondary antibody was added and incubated at room temperature for 1 h. Exposure was performed using an ultrasensitive multifunctional imager and subsequent analysis was performed by ImageJ software.

### 2.8 Immunofluorescence

Discard medium from cells in the logarithmic growth phase, add PBS slowly to the rim of the dish and wash 2 times for 5 s each time. Add 4% paraformaldehyde fixative, 4 °C, and fix for 2 h. Afterwards, using TBS buffer that was cold at 4 °C, rinse 3 times for 5 min each. The fixed cells were permeabilised using 0.3% Triton X-100 solution to ensure that the antibody could reach the antigen site. The cell crawls were closed with a solution containing 1% BSA. Afterwards, the primary and secondary antibodies were incubated according to the recommended dosage in the antibody instructions. Fixation was blocked with a discolouring reagent containing DAPI, sealed with neutral resin and finally analysed using ImageJ software. HUVECs were treated with CLY at a concentration of 0.5 mg/mL in this experiment.

### 2.9 Cell scratch assay

HUVEC cells were inoculated in six-well plates at a concentration of 2 × 10^5^/mL. When the cell number density was 95%, the cell scratching experiment was performed. A sterilised ruler and a marker pen were used to mark the back of the six-well plate. A pipette tip was used to make a scratch perpendicular to the bottom of the six-well plate. Subsequently, along the walls of the wells, PBS was added slowly and the cells were washed three times for 5 s each time. After incubation for 0 and 24 h, cell growth was observed using a light microscope and pictures were taken. The percentage of migrate cells was calculated using the formula: migration cell number (%) = (0 h wound width - 24 h/48 h wound width)/0 h wound width × 100%.

### 2.10 qPCR

Total RNA was rapidly isolated from cells using the Rapid RNA Extraction Kit (R711-01/02, Vazyme) and following its instructions. Afterwards, the initial cDNA strand was synthesised using the Reverse Transcription Kit (HiScript II Q Select RT SuperMix, Vazyme) and following the instructions. The mRNA expression levels of target genes were quantified using the ChamQ SYBR qPCR Master Mix kit (Q321-02, Vazyme) and data were analysed. the GAPDH gene was used as an internal control in the experiments. In this study, HUVEC and THP-1 cells were treated with CLY at concentrations of 0.5 mg/mL and 0.4 mg/mL, respectively. The primer sequences are listed in Table 2.

**TABLE 2:** Sequences of the primers designed for RT-qPCR.

Gene	Forward sequence	Reverse sequence
CD31	TGA​CCC​TTC​TGC​TCT​GTT​CAA	CTG​AGG​CTT​GAC​GTG​AGA​GG
VEGF	GCA​GAA​TCA​TCA​CGA​AGT​GGT	CCA​GGG​TCT​CGA​TTG​GAT​GG
IL-6	CAC​TGG​CAG​AAA​ACA​ACC​TGA	GAT​TTT​CAC​CAG​GCA​AGT​CTC​C
IL-1β	TGA​TGG​CTT​ATT​ACA​GTG​GCA​A	TAG​TGG​TGG​TCG​GAG​ATT​CG
TNF-α	TGC​ACT​TTG​GAG​TGA​TCG​GC	ACT​CGG​GGT​TCG​AGA​AGA​TG
NF-KB	GCC​TCC​ACA​AGG​CAG​CAA​ATA	CAC​CAC​TGG​TCA​GAG​ACT​CGG​TAA
GAPDH	GCA​CCG​TCA​AGG​CTG​AGA​AC	TGG​TGA​AGA​CGC​CAG​TGG​A

### 2.11 CCK-8

HUVEC and THP-1 cells were inoculated into 96 wells at a density of 4 × 10^4^ and later treated with different concentrations of CLY (mg/mL) and after 24 h of treatment, the absorbance in each test well was detected at 450 nm using an enzyme marker. Based on the results, the maximum non-cytotoxic concentration (with cell viability >80%) was selected for the subsequent functional experiments to ensure that the observed effects were due to specific biological modulation. Concentrations of CLY 0.5 mg/mL and 0.4 mg/mL were ultimately selected for HUVECs and THP-1 cells, respectively, and were used in all subsequent functional experiments.

### 2.12 Flow cytometry instrument

THP-1 cells were cultured in a 5% CO_2_ incubator using high-glucose DMEM for 24 h. THP-1 cells were induced using PMA (100 ng/mL) for 48 h to induce differentiation into macrophages and then stimulated with LPS (1 μg/mL) for 4 h after induction. Finally, intervention using CLY (0.4 mg/mL) was performed for 48 h. After the intervention was completed, the cells were washed 3 times using PBS to make a cell suspension. To the cell suspension, iNOS (iNOS-APC, 696807, Biolegend) and CD206 (CD206-PE, 141705, Biolegend) antibody were added and treated for 30 min in the dark. Treatments were completed washed using PBS and analysed using flow cytometry.

### 2.13 Statistical analyses

All experimental data were analysed using GraphPad 9.0 and expressed as mean ± standard error of the mean (SEM). In addition, analysis of variance (ANOVA) was used to test the significance of data conforming to a normal distribution. For multi-group comparisons, one-way ANOVA with Tukey’s honestly significant difference (HSD) *post hoc* test was uniformly applied to determine inter-group significance. Significance between the two groups was assessed using the independent samples t-test. p-values less than 0.05 were considered statistically significant.

## 3 Results

### 3.1 Metabolites in CLY

**FIGURE 2:**
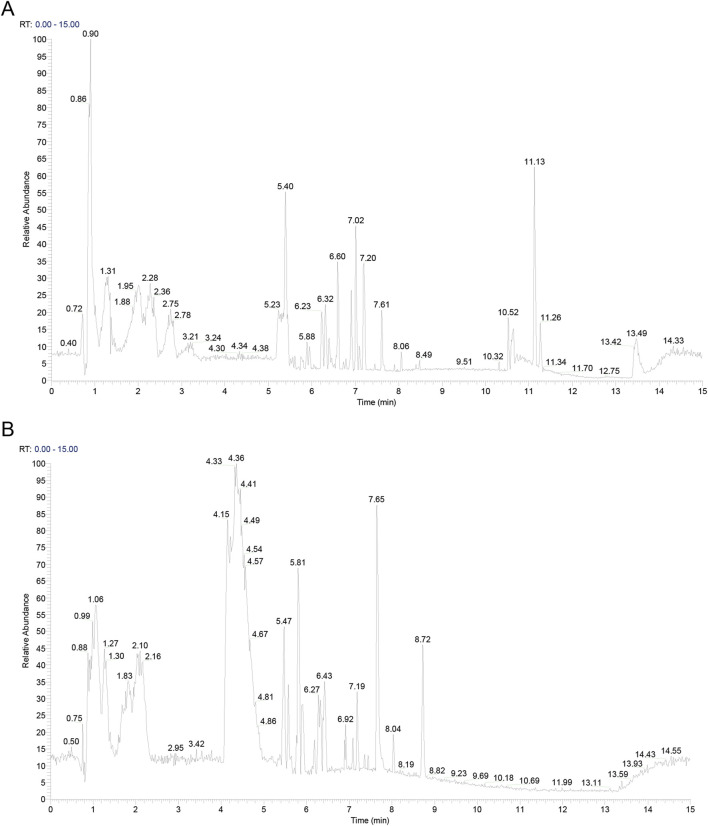
Metabolites in CLY. **(A)** Positive ion mode mapping. **(B)** Negative ion mode mapping.

The active metabolites in CLY were analysed using UHPLC-MS/MS analysis technique and a total of 357 metabolites were identified. The metabolites of CLY species were preliminarily identified by metabolite structure identification using exact mass number matching and secondary spectral matching, and searching the TCM database (Figure 2). Table 3 lists the molecular formulas, identified metabolite, adduct and m/z of the top 30 metabolites according to the mzCloud best match organization. The full list of all 357 identified metabolites, along with their proposed identification details, is provided in Supplementary Table S1.

**TABLE 3:** MS data and identification results of metabolites of CLY (top50).

No.	tR/min	Identified component	Formula	Adduct	m/z
1	0.876267	Arginine	C6H14N4O2	[M + H]+	175.1189
2	0.87955	Bergapten	C12H8O4	[M-H]-	215.0317
3	0.87955	Arabinonic Acid	C5H10O6	[M-H]-	165.0388
4	0.87955	Gluconic Acid	C6H12O7	[M-H]-	195.0498
5	0.893483	Glucose	C6H12O6	[M-H]-	179.0545
6	0.9041	Choline	C5H14NO	[M]+	104.1072
7	0.945783	Betaine	C5H11NO2	[M + H]+	118.0865
8	0.95975	Proline	C5H9NO2	[M + H]+	116.0708
9	0.95975	Trigonelline	C7H7NO2	[M + H]+	138.055
10	0.963133	Malic Acid	C4H6O5	[M-H]-	133.0126
11	0.987617	D-Pipecolic Acid	C6H11NO2	[M + H]+	130.0862
12	0.991133	Malonic Acid	C3H4O4	[M-H]-	103.0019
13	1.0155	Beta-D-Glucose	C6H12O6	[M + NH4]+	198.0972
14	1.046767	Citric Acid	C6H8O7	[M-H]-	191.0185
15	1.046767	Galactose	C6H12O6	[M-H]-	179.0545
16	1.265767	Valine	C5H11NO2	[M + H]+	118.0865
17	1.279617	Pyroglutamic Acid	C5H7NO3	[M + H]+	130.0499
18	1.3959	Shikimic Acid	C7H10O5	[M-H]-	173.044
19	1.418317	Pipecolic Acid	C6H11NO2	[M + H]+	130.0861
20	1.584617	Gamma-Guanidinobutyric Acid	C5H11N3O2	[M + H]+	146.0921
21	1.6124	Cytidine	C9H13N3O5	[M + H]+	244.0922
22	1.7373	Adenine	C5H5N5	[M + H]+	136.0574
23	2.0572	Nicotinamide	C6H6N2O	[M + H]+	123.0555
24	2.2518	Piperidine	C5H11N	[M + H]+	86.0969
25	2.279567	Isoleucine	C6H13NO2	[M + H]+	132.1018
26	2.75205	Tyrosine	C9H11NO3	[M + H]+	182.081
27	2.821517	2,3-Dihydrobenzofuran	C8H8O	[M + H]+	121.0649
28	2.903433	1,2,4-Benzenetriol	C6H6O3	[M-H]-	125.0228
29	3.37775	2-Pyrrolidone	C4H7NO	[M + H]+	86.06053
30	4.355367	Gallic Acid	C7H6O5	[M-H]-	169.0127
31	4.355367	Methyl 2-Furoate	C6H6O3	[M-H]-	125.0228
32	5.368017	Adenosine	C10H13N5O4	[M + H]+	268.1032
33	5.395817	Phenylalanine	C9H11NO2	[M + H]+	166.0862
34	5.395817	Phenylethanolamine	C8H11NO	[M + H-H2O]+	120.0808
35	5.521283	Pyrogallol	C6H6O3	[M + H]+	127.0391
36	5.898167	Tetrahydroharman-3-Carboxylic Acid	C13H14N2O2	[M + H]+	231.1124
37	5.907217	Gallotannin	C27H24O18	[M-H]-	635.0894
38	6.233783	Isoquercitrin	C21H20O12	[M + H]+	465.1033
39	6.315017	Ellagic Acid	C14H6O8	[M-H]-	300.9977
40	6.3176	Liquiritin	C21H22O9	[M + H]+	419.134
41	6.907467	Decursinol	C14H14O4	[M + H]+	247.0961
42	6.907467	Tinnevellin Glucoside	C20H24O9	[M + H]+	409.1494
43	6.91955	Genistin	C21H20O10	[M-H]-	431.0968
44	7.020216	7-Methoxy-4-Methylcoumarin	C11H10O3	[M + H]+	191.0703
45	7.088167	Quercetin	C15H10O7	[M-H]-	301.0348
46	7.200583	Endocrocin	C16H10O7	[M-H]-	313.0345
47	7.202267	Gastrodin	C13H18O7	[M + Na]+	309.0867
48	8.04915	Rhein	C15H8O6	[M-H]-	283.0245
49	8.723233	Emodin	C15H10O5	[M-H]-	269.0452
50	11.12915	Oleamide	C18H35NO	[M + NH4]+	282.279

### 3.2 CLY increases indicators of wound neovascularization to accelerate wound healing in DU rats

**FIGURE 3:**
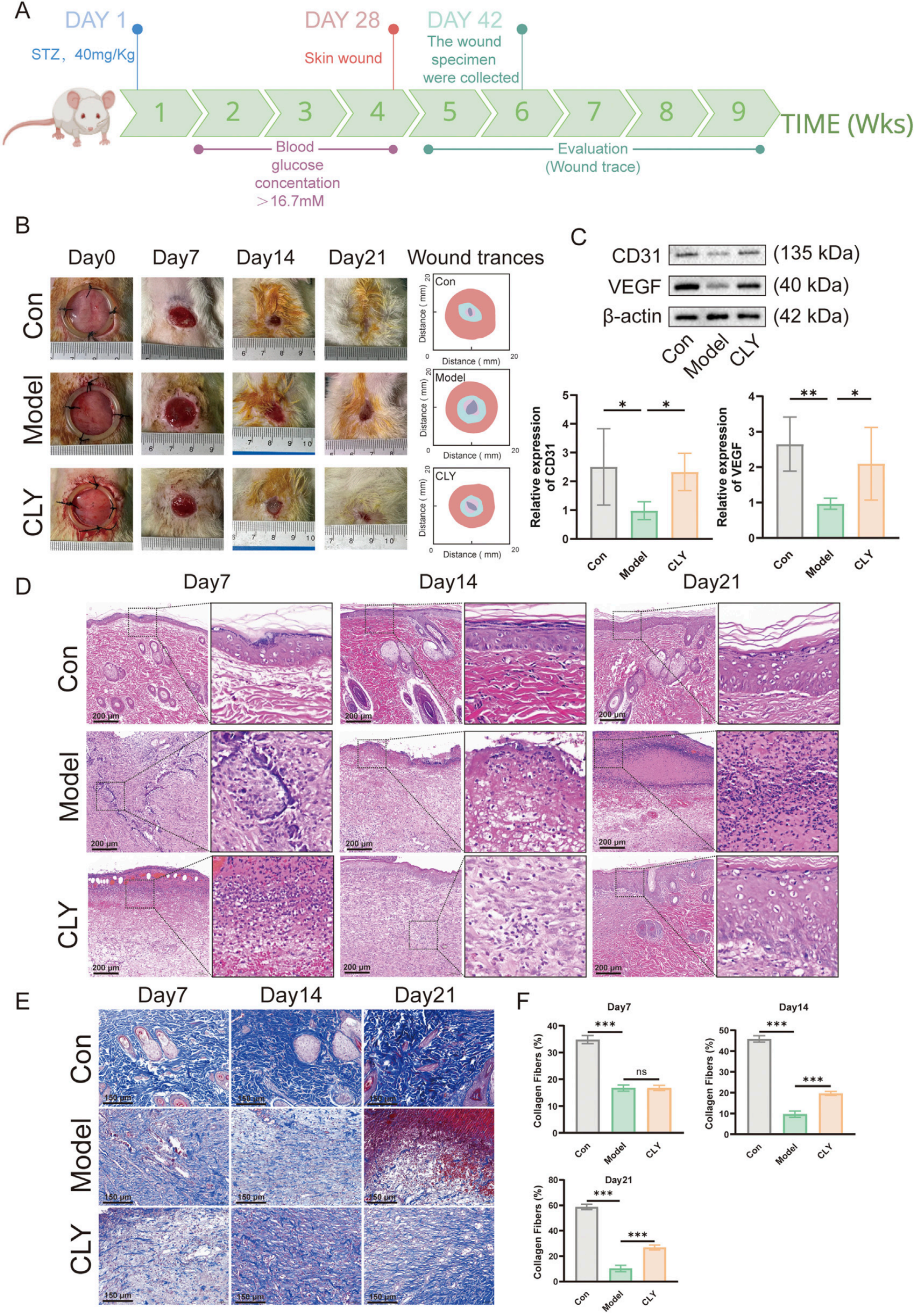
Effect of CLY on wound healing in rats with DUs. **(A)** Time points of wound healing observation in diabetic rats. **(B)** Representative images of rat skin wounds at different time points post-repair and the corresponding trace of wound-bed closure. Red, day 0; blue, day 7; pink, day 14; gray, day 21. **(C)** Expression levels of CD31 and VEGF in the trabecular tissues of rats in the three groups (n = 6) on the seventh day. **(D)** HE staining analysis of the traumatic tissues of rats in the three groups. **(E)** Masson staining analysis of rats in three groups. **(F)** Quantitative analysis of Masson staining in three groups of rats (n = 6). Compared with the model group, ^*^P < 0.05, ^**^P < 0.01, ^***^P < 0.001, all data were expressed as mean ± SEM.

In this study, the pathological process of DU wounds observed in humans was mimicked by first inducing the formation of DM in rats by STZ and later by surgically creating open wounds on the back of the DM rat model. After the creation of DU wounds, four wound observation time points were selected to assess the wound status (Figure 3A). The wound area over time indicated slower healing in the diabetic model group and a significantly improved healing rate in the CLY-treated group, compared to normal controls (Figure 3B). Subsequently, the tissue was collected on the seventh day of each group and tested for neovascularization. Western blot showed a significant increase in the neovascularization indices of CD31 and VEGF in the CLY group compared to the control group. In contrast to the model group, no significant improvement was observed in the neovascularization indexes (Figure 3C). Subsequently, we carried out pathological staining analysis of the wound tissue, and in HE staining, we found that CLY could significantly reduce the aggregation of inflammatory cells in the wound tissue, and reduce the inflammatory response of the wound. In Masson staining, with the increase of time, collagen fibre tissues in the wound tissue of the rats intervened by CLY significantly increased (Figures 3D–F). These evidences suggest that CLY can effectively increase the angiogenic profile of wound tissue and accelerate wound healing.

### 3.3 Network pharmacology analysis of the mechanism of CLY in the treatment of DU

**FIGURE 4:**
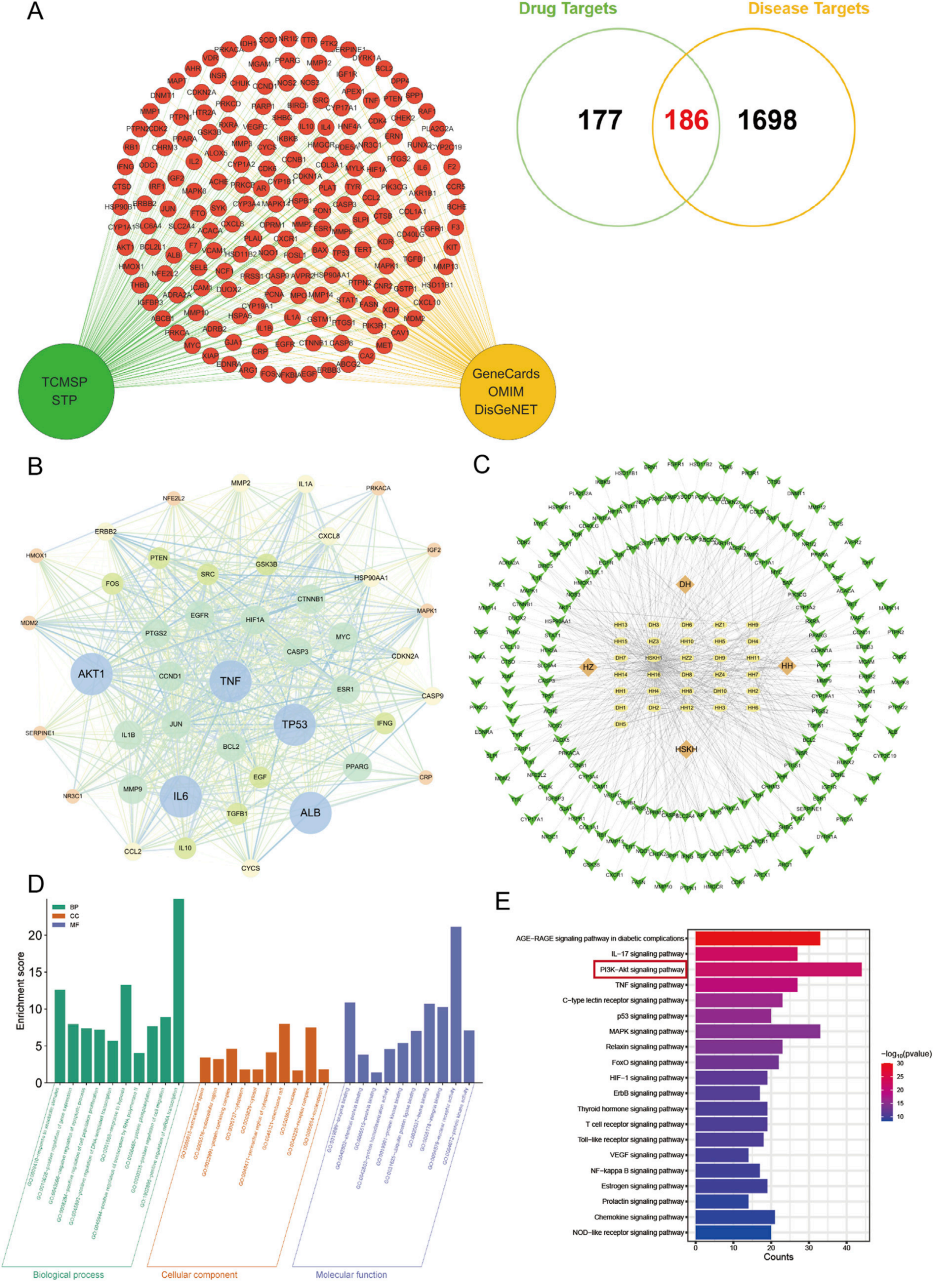
Network pharmacological analysis of CLY for DU. **(A)** 186 potential targets of CLY for the treatment of DUs were demonstrated by Venn diagram. **(B)** Protein-protein interaction network analysis. The top 44 targets in terms of degree value were visualised. **(C)** Metabolite-target-pathway-disease network diagram of CLY. Green arrows represent disease targets, yellow ellipses denote metabolites in CLY, and orange diamonds indicate the abbreviation of the botanical drugs. **(D,E)** GO and KEGG enrichment analysis, with the PI3K-Akt signaling pathway being the most significant in the enrichment.

We performed drug target screening by using TCMSP database and SwissTargetPrediction database, and finally obtained 363 drug targets. By searching Genecards database, OMIM database and DisgeNet database, the total of 1884 was obtained after integration. The two were plotted on a Venn diagram, and the number of intersecting disease-drug common targets of the two was 186, which were potential targets for CLY treatment of DU (Figure 4A).

The 186 drug and disease common targets were imported using the STRING database, see later Protein-Protein Interaction (PPI) Network. Interactions between these targets were analysed using Cytoscape 3.6.1. Based on the target-to-target degree values, 44 targets were selected and visualised in the network diagram (Figure 4B). Among the 44 targets, the top 5 targets were TNF, IL-6, AKT1, ALB, TP53. Table 4 lists the Target name, Degree, Betweenness and Closeness of the top 5 targets according to the ranking of the degree value.

**TABLE 4:** Core therapeutic targets of CLY for diabetic foot ulcer treatment.

Gene symbol	Target name	Degree	Betweenness	Closeness
TNF	tumor necrosis factor	154	1359.580398	0.00462963
IL6	interleukin- 6	152	1197.133306	0.004587156
AKT1	AKT serine/threonine kinase 1	148	1193.154662	0.004504505
ALB	Albumin	146	1228.96016	0.004464286
TP53	Tumor protein p53	141	885.1681608	0.004366812

In order to further understand the interactions among active metabolites, core targets and pathways of CLY, the above data were imported into Cytoscape 3.6.1 and a ‘metabolite-target-pathway-disease’ network diagram was constructed (Figure 4C).

A total of 186 common targets were identified for GO and KEGG enrichment analysis. KEGG enrichment analysis showed that a total of 20 signaling pathways directly or indirectly affected DU, including AGE-RAGE signaling pathway in diabetic complications, IL-17 signaling pathway and PI3K-Akt signaling pathway. PI3K-Akt signaling pathway and TNF signaling pathway. Subsequently, we performed follow-up experiments to validate the PI3K-Akt signaling pathway, which was the highest rated pathway among these pathways. GO enrichment analysis showed that the therapeutic mechanisms of CLY in DU diseases mainly involved the regulation of apoptosis, cell population proliferation, and positive regulation of DNA-triggered transcription. Overall, these findings all suggest that CLY treatment of DU works through multiple metabolites and pathways, among which the PI3K-Akt signaling pathway is the most significant in the enrichment, which is likely to be the main signaling pathway of CLY treatment of DU (Figures 4D,E).

### 3.4 CLY ameliorates high glucose-induced HUVEC dysfunction

**FIGURE 5:**
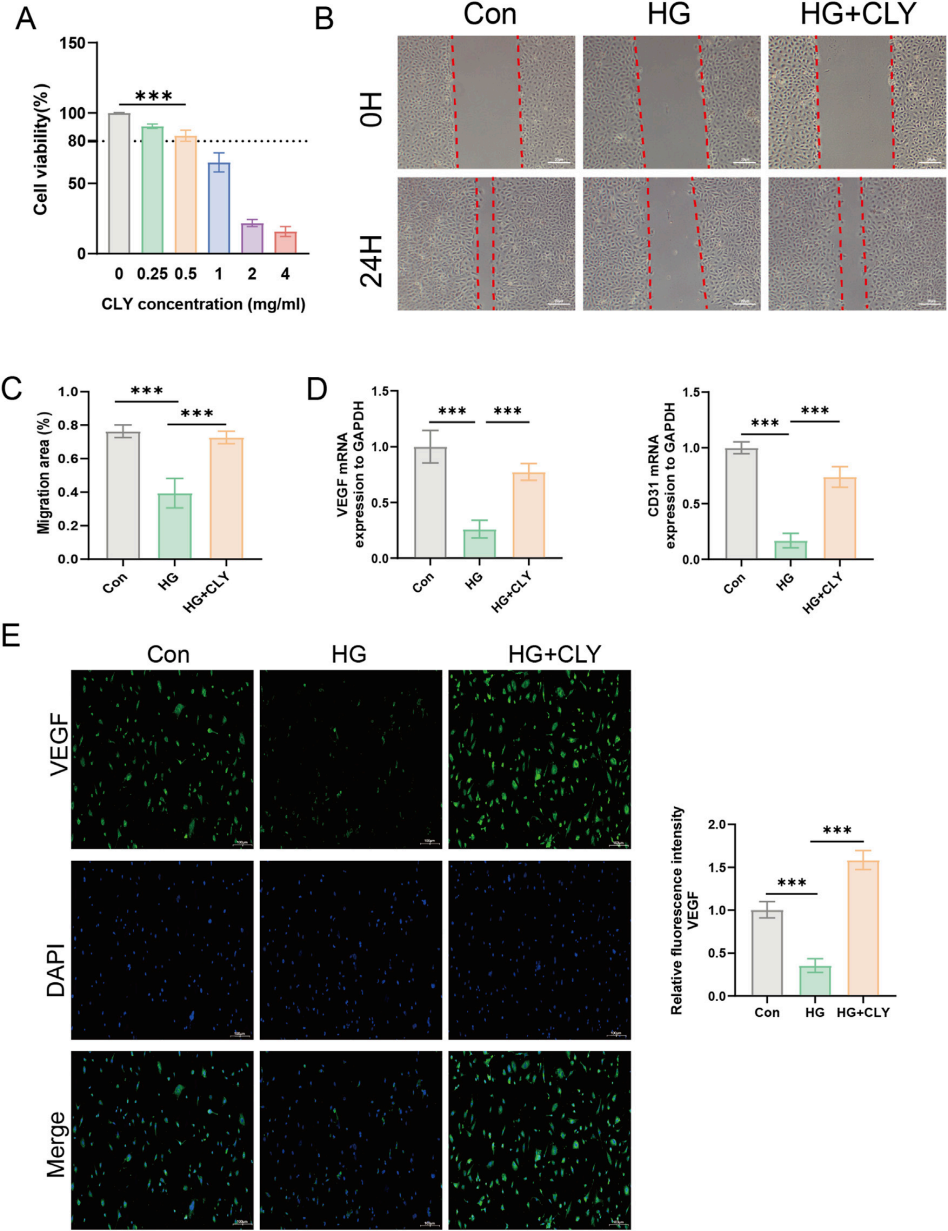
Effect of CLY on high glucose-induced HUVEC cells. **(A)** CCK-8 assay to determine the effect of CLY on HUVEC cell proliferation. Compared with the 0 mg/mL CLY group, ^***^P < 0.001. **(B)** Cell scratch assay to observe the effect of CLY on HUVEC cell migration (band = 20 μM). **(C)** Quantitative analysis of scratch area. **(D)** PCR assay to analyse the mRNA expression of VEGF and CD31 in different subgroups (n = 3). **(E)** Cellular immunofluorescence assay to detect VEGF expression (band = 100 μM). Compared with the HG group (25 mM), ^***^P < 0.001, all data were expressed as mean ± SEM. HUVECs were treated with CLY at a concentration of 0.5 mg/mL in this experiment.

Through network pharmacological studies, we predicted that CLY its likely to improve DU through the PI3K-Akt signaling pathway. To further validate its mechanism of action, we assessed its efficacy by inducing HUVEC cells by high glucose *in vitro* to mimic the traumatic environment. Using CCK-8 assay, we examined the effect of CLY on HUVEC proliferation at different concentrations. We observed that CLY concentrations below 0.5 mg/mL did not affect the proliferation of HUVEC cell lines. And when the CLY concentration exceeded 0.5 mg/mL, a significant decrease in HUVEC cell viability could be observed. In view of the above results, we chose a concentration of 0.5 mg/mL for subsequent experiments (Figure 5A). Next, we tested the improvement of CLY on the migration ability of high glucose (HG)-treated (25 mM) HUVEC by cell scratch assay. The results showed that the migration ability of HUVEC induced by HG was significantly decreased compared with the control group (normal glucose, 5 mM), while the migration ability of HUVEC cells was significantly increased after CLY treatment (0.5 mg/mL) (Figures 5B,C). Subsequently, we further examined the expression of mRNA in HUVEC after different treatments, and we found that VEGF and CD31 were significantly higher in the control group (normal glucose, 5 mM) compared to the HG group and in the CLY group (Figure 5D). VEGF immunofluorescence experiments further confirmed this result. These results all suggest that high glucose reduces angiogenesis in HUVEC, and the use of CLY can reverse this result (Figure 5E).

### 3.5 CLY ameliorates high glucose-induced HUVEC cell dysfunction through the PI3K-AKT signaling pathway

**FIGURE 6:**
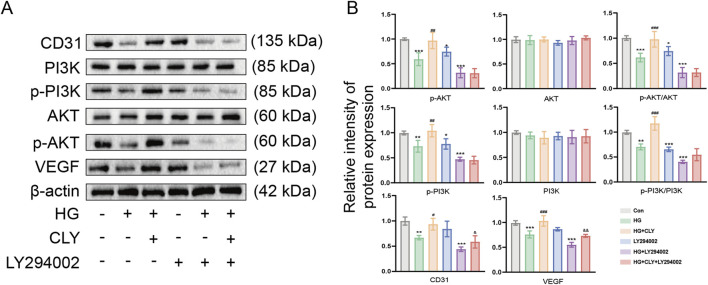
CLY ameliorates high glucose-induced HUVEC cell dysfunction via PI3K-Akt signaling pathway. **(A)** Representative Western blot of CLY on PI3K/Akt pathway protein expression in high glucose-induced HUVEC cells after LY294002 treatment. **(B)** Quantitative analysis of the relative protein expression levels of the PI3K/Akt signaling pathway in CLY and LY294002 treated high glucose-induced HUVEC cells (n = 3). Compared with the control group (normal glucose, 5 mM), ^*^P < 0.05, ^**^P < 0.01, ^***^P < 0.001. Compared with the HG group, ^#^P < 0.05, ^##^P < 0.01, ^###^P < 0.001. Compared with the HG + LY294002 group, ^&^P < 0.05, ^&&^P < 0.01, all data were expressed as mean ± SEM. HUVECs were treated with CLY at a concentration of 0.5 mg/mL in this experiment.

To further explore the mechanism by which CLY improves high glucose-induced HUVEC dysfunction, we used LY294002 to examine the effects of CLY on the PI3K-Akt signaling pathway (Figures 6A,B). The results showed that HG-treated (25 mM) HUVEC, compared with the control group (normal glucose (5 mM) without any treatment), showed a significant reduction in key proteins (p-PI3K and p-AKT) in the PI3K-Akt signaling pathway. However, HUVEC treated with CLY-intervention HG (25 mM) showed increased expression of p-PI3K and p-AKT, along with significantly higher angiogenesis-related proteins.

After LY294002 treatment of HUVEC, the expression of p-PI3K and p-AKT was significantly decreased, along with angiogenesis-related indexes, compared with the control group (normal glucose (5 mM) without any treatment). After HG + LY294002 treatment, the expression of related proteins decreased even more significantly compared with the control group (normal glucose (5 mM) without any treatment). However, HUVEC treated with HG + CLY + LY394002 showed a slight increase in the expression of angiogenesis-related proteins CD31 and VEGF were both increased to different degrees compared with the HG + LY294002 group. All these results demonstrated that CLY could reverse the dysfunction of HG-treated HUVEC.

### 3.6 CLY ameliorates the inflammatory response of THP-1 cells induced by LPS

**FIGURE 7:**
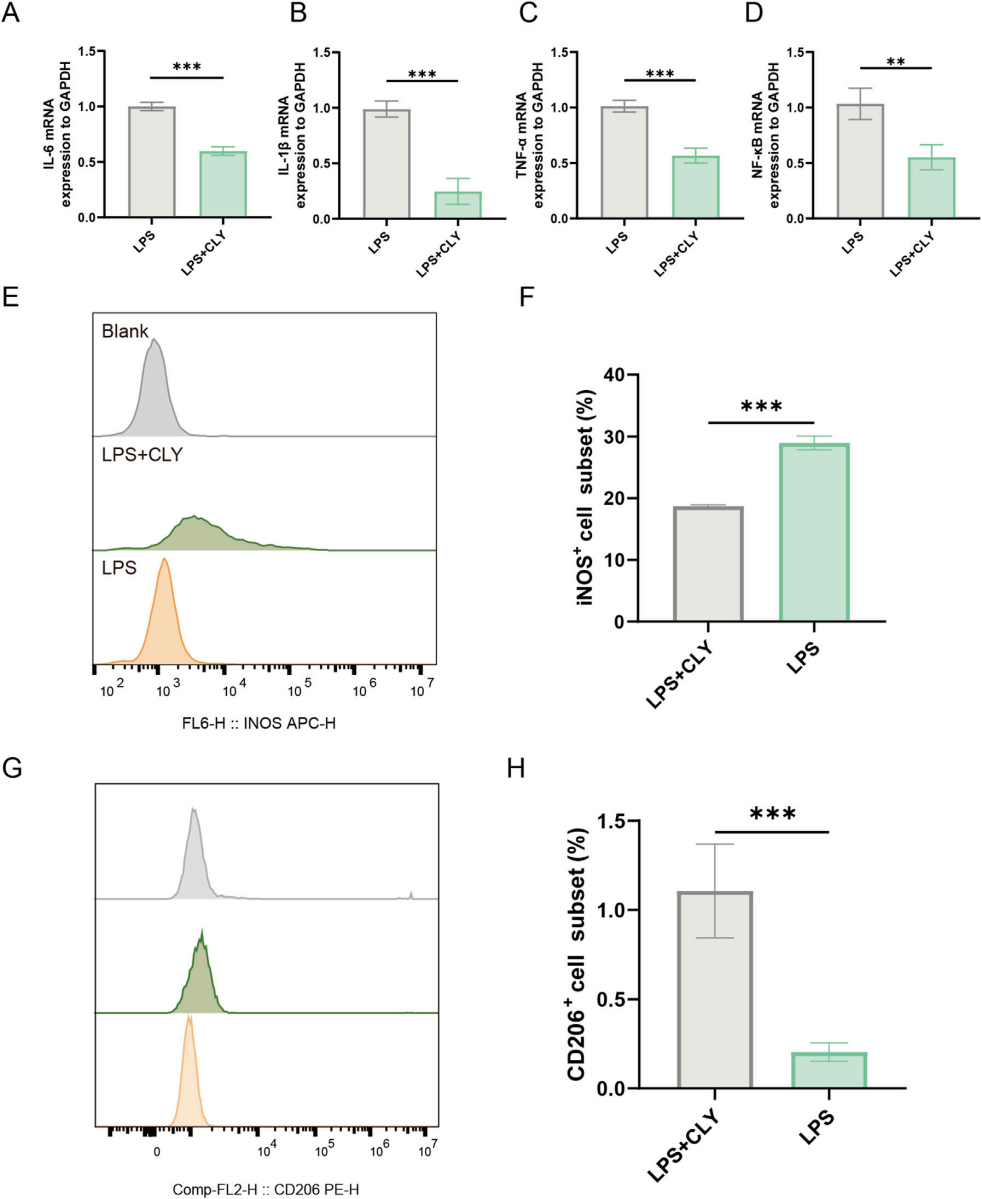
CLY ameliorates the inflammatory response of THP-1 cells induced by LPS. **(A–D)** PCR analysis was performed to detect IL-6, IL-1β, TNF-α, and NF-κB mRNA expression (n = 3). **(E,F)** Assessment of M1-type macrophage counts using flow cytometry revealed a significant decrease in M1-type macrophage counts after CLY treatment (n = 3). **(G,H)** Assessment of M2-type macrophage counts using flow cytometry revealed a significant increase in M2-type macrophage counts after CLY treatment (n = 3). Compared with the LPS group, **P < 0.01, ***P < 0.001, all data were expressed as mean ± SEM. THP-1 cells were treated with CLY at a concentration of 0.4 mg/mL in this experiment.

Wound healing is a complex process that involves local angiogenesis, improvement of wound inflammation and fibroblast aggregation in the wound. And in the inflammation of wounds, the polarization of M1 macrophages plays a key role in maintaining the inflammatory response of wounds. To further explore the effect of CLY on wound inflammation and to explore its potential in wound healing. We assessed its effect on THP-1. The effect of CLY on THP-1 proliferation was assessed using CCK-8 assay across varying concentrations. Exposure to CLY below 0.4 mg/mL showed no significant impact on THP-1 cell proliferation, whereas concentrations exceeding 0.4 mg/mL resulted in markedly reduced cell viability. Consequently, 0.4 mg/mL CLY was selected for subsequent experimental procedures (Supplementary Figure S1). We used LPS to induce THP-1 cells and intervened with CLY (0.4 mg/mL) to mimic the anti-inflammatory process of CLY in DU. The results showed that mRNA levels of inflammatory factors IL-6, IL-1β, TNF-α, and NF-κB were significantly reduced after CLY intervention compared to LPS (Figures 7A–D). We also examined the number of M1 and M2-type macrophages. The results showed that compared with LPS stimulation, CLY treatment reduced the proportion of M1-type macrophages while increasing the population of M2-type macrophages (Figures 7E–H). All these results demonstrated that CLY could exert anti-inflammatory effects by modulation of macrophage polarization and reducing the secretion of inflammatory factors.

## 4 Discussion

As one of the most common and serious complications of diabetes, the clinical management of DU is often very challenging. Focusing on CLY, a traditional Chinese medicine compound, this study systematically revealed its potential mechanism for promoting DU healing by integrating modern pharmacological techniques with experimental validation, providing more possibilities for the clinical treatment of DU.

In this study, we first verified the effect of CLY by constructing a diabetic rat model. We found that CLY significantly accelerated wound healing in STZ-induced diabetic rats compared with the control group and significantly increased the levels of CD31 and VEGF in the sore tissues. VEGF belongs to the family of cytokine growth factors, which is an important mediator of vascular permeability and angiogenesis, and it promotes vascular growth, collagen deposition and epithelialization in the wound healing process (Zubair and Ahmad, 2019). CD31 is considered to be a key factor in the evaluation of neovascularization, acting as a pro-angiogenic signal by co-operating with VEGF (Martin and Leibovich, 2005). These findings imply that CLY is effective in increasing the angiogenic profile of wound tissue and accelerating wound healing. Subsequently, we performed compositional analysis of CLY by UHPLC-MS/MS technology and identified a total of 357 metabolites. Further screening by network pharmacology revealed 186 intersections of CLY potential targets of action with DU disease targets, with TNF, IL-6, AKT1, ALB, and TP53 as core targets. These genes play a key role in the treatment of DU with CLY. TNF is a core cytokine in the inflammatory response, which can be driven either directly by inducing the expression of inflammatory genes or indirectly by triggering an inflammatory immune response (Van Loo and Bertrand, 2023). IL- 6 is an important inflammatory marker, and some studies have found that impairment of the IL-6 signaling pathway leads to delayed wound healing (Johnson et al., 2020; Lin et al., 2003). AKT1 is a key molecule in the cell signaling pathway, which has been shown to play an important role in the regulation of macrophage activation and M1/M2 polarization (Verga et al., 2017).

KEGG enrichment analysis showed that these intersecting genes were significantly enriched in the PI3K-Akt signaling pathway, AGE-RAGE signaling pathway and IL-17 signaling pathway, with the PI3K-Akt signaling pathway being the most significantly enriched. PI3K-Akt signaling pathway is involved in a variety of physiological processes, and it is an important signaling pathway for the control of cell survival, apoptosis and differentiation (Wang et al., 2022; Jafari et al., 2019). This pathway plays an equally important role in the regulation of macrophage status and in response to inflammatory signals (Verga et al., 2017). Combined with the identification results from UHPLC-MS/MS technology and literature review, the key metabolites identified–such as gallic acid, emodin, and quercetin–were found to directly or indirectly influence the PI3K-Akt signaling pathway. Quercetin can reduce the activation of pro-inflammatory cytokines in diabetic wounds, and the PI3K-Akt signaling pathway plays an important regulatory role in this process (Zhang et al., 2024). Kant’s research found that quercetin increased the expression of IL-10, VEGF, and TGF-β1 in granulation tissue/healing tissue of diabetic wounds, while reducing the expression of TNF-α, IL-1β, and MMP-9, effectively improving the repair and regeneration of diabetic skin wounds in rats (Kant et al., 2021). Gallic acid accelerated the cell migration of keratinocytes and fibroblasts under normal and high sugar conditions, and promoted wound healing under normal and high sugar conditions (Yang et al., 2016). Emodin can accelerate tissue healing of diabetic wounds and reorganize the local microenvironment by increasing the number of anti-inflammatory M2 macrophages and promoting the synthesis of extracellular matrix (ECM) in fibroblasts (Yang et al., 2024). It has been found that activation of the PI3K-Akt signaling pathway induces anti-inflammatory cytokine expression, promotes an M2-like phenotype, and facilitates tissue repair and inflammatory regression. Conversely, inhibition of PI3K-Akt signaling pathway enhances the M1-like phenotype (Yang et al., 2023). Activation of the PI3K-Akt signaling pathway is also important for angiogenesis (Yao et al., 2024). It has been found that activation of the PI3K-Akt signaling pathway increases VEGF secretion and regulates the expression of other angiogenic factors, such as nitric oxide and angiopoietin (Karar and Maity, 2011). Our *in vitro* experiments revealed that CLY reversed high glucose-induced HUVEC dysfunction and promoted cell migration and angiogenic protein expression, and this effect could be partially counteracted by the inhibitor LY294002. These results clarify the regulatory role of the PI3K-Akt signaling pathway. Meanwhile, *in vitro* experiments using HUVECs demonstrated that CLY activates Akt signaling, a finding consistent with the identification of AKT1 as a core hub target in the network pharmacology analysis.

CLY may exhibit pleiotropic effects, as evidenced by the concurrent enrichment of the AGE-RAGE and IL-17 signaling pathways in the network pharmacology analysis. The PI3K-Akt pathway was selected as the focus here due to its high ranking in the enrichment results. Its downstream mechanisms may encompass eNOS activation, Akt-mediated endothelial cell survival signaling, or the modulation of the wound inflammatory response via Akt’s regulation of NF-κB activity. Activated Akt directly phosphorylates the Ser1177 residue of eNOS, leading to its activation and subsequent production of nitric oxide (NO). NO stimulates endothelial cell proliferation, migration, and neovascularization, which constitutes the foundation of granulation tissue formation (Hu et al., 2021). Furthermore, as an upstream activator of NF-κB, Akt typically promotes its mediated sustained inflammatory response by phosphorylating and relieving the inhibition of NF-κB (Li et al., 2025). CLY appears to alleviate this sustained inflammatory response by inhibiting this process.

Inflammatory response plays a key role in diabetic wound healing (Lv et al., 2023). High levels of inflammatory cytokines and persistent chronic inflammation of predominantly M1 macrophages result in delayed wound healing (Sharifiaghdam et al., 2022). By shifting macrophages from the M1 phenotype (pro-inflammatory state) to the M2 phenotype (anti-inflammatory state) it is possible to ameliorate the inflammatory situation and reduce the production of pro-inflammatory cytokines (Louiselle et al., 2021). Our study found that CLY significantly decreased the expression of the M1-type macrophage marker iNOS while upregulating the level of the M2-type marker CD206 in the LPS-induced THP-1 cell inflammation model. Meanwhile, qPCR results showed that CLY inhibited the expression of pro-inflammatory factors IL-6, IL-1β, TNF-α and NF-κB mRNA. The inflammatory markers detected in this study are consistent with the hub targets TNF and IL-6 predicted by network pharmacology. These results suggest that CLY synergistically improves the DU trauma microenvironment by promoting macrophage polarization towards M2-type and inhibiting the inflammatory cascade response. However, it should be noted that the anti-inflammatory effects of CLY in DUs proposed in this study are based primarily on mRNA expression data. The observed downregulation of pro-inflammatory cytokine genes (IL-6, TNF-α), coupled with the shift in macrophage polarization toward the M2 phenotype, suggests that CLY may inhibit NF-κB pathway activation. This effect is potentially mediated through PI3K-Akt pathway-dependent mechanisms or alternative pathways, as supported by our network pharmacology analysis. Therefore, definitive confirmation of key cytokine proteins and the activation status of NF-κB would be a valuable next step to further validate these findings.

In this study, we systematically revealed the multi-target mechanism of CLY in the treatment of DU through modern pharmacological techniques and experimental validation. The PI3K-Akt pathway appears central, but other pathways (inflammatory cascades, *etc.*) are also modulated as indicated by the network analysis and multi-target nature of CLY. CLY can promote neovascularization by activating the PI3K-AKT pathway and up-regulating the expression of VEGF/CD31. However, inhibitors were not administered in the animal models. The *in vivo* mechanism was extrapolated from the *in vitro* results, suggesting that CLY may mediate its effects through pathways such as PI3K- Akt. In addition, CLY can regulate macrophage polarization and inhibit the release of inflammatory factors such as IL-6 and TNF-α to improve the microenvironment of the sore. While our current study was focused on elucidating CLY overall mechanism in promoting DU healing, it did not include experimental validation of individual metabolites. Subsequent research will involve targeted experimental analyses of these specific metabolites to further substantiate the material basis underlying CLY therapeutic action in DU treatment.

## 5 Conclusion

In conclusion, the mechanism of action by which CLY can promote the healing of DU was identified in our study. In this study, CLY could promote high glucose-induced HUVEC dysfunction *in vitro* by regulating the PI3K-Akt signaling pathway. Our data suggest that CLY can promote elevated expression related to wound angiogenesis, which in turn accelerates wound healing in a STZ-induced diabetic rat model. In addition, CLY inhibited M1 polarization of macrophages and reduced inflammatory factor expression. This study confirms the therapeutic potential of CLY for DUs and elucidates its underlying mechanisms.

## Data Availability

The original contributions presented in the study are included in the article/Supplementary Material, further inquiries can be directed to the corresponding authors.

## References

[B1] Armstrong D. G. Tan T.-W. Boulton A. J. M. Bus S. A. (2023). Diabetic foot ulcers: a review. JAMA 330, 62–75. 10.1001/jama.2023.10578 37395769 PMC10723802

[B2] Deng H. Li B. Shen Q. Zhang C. Kuang L. Chen R. (2023). Mechanisms of diabetic foot ulceration: a review. J. Diabetes 15, 299–312. 10.1111/1753-0407.13372 36891783 PMC10101842

[B3] Hao D. C. Xiao P. G. (2014). Network pharmacology: a Rosetta Stone for traditional Chinese medicine. Drug Dev. Res. 75, 299–312. 10.1002/ddr.21214 25160070

[B4] Hu Y. Tao R. Chen L. Xiong Y. Xue H. Hu L. (2021). Exosomes derived from pioglitazone-pretreated MSCs accelerate diabetic wound healing through enhancing angiogenesis. J. Nanobiotechnology 19, 150. 10.1186/s12951-021-00894-5 34020670 PMC8139165

[B5] Jafari M. Ghadami E. Dadkhah T. Akhavan‐Niaki H. (2019). PI3k/AKT signaling pathway: erythropoiesis and beyond. J. Cell Physiol. 234, 2373–2385. 10.1002/jcp.27262 30192008

[B6] Johnson B. Z. Stevenson A. W. Prêle C. M. Fear M. W. Wood F. M. (2020). The role of IL-6 in skin fibrosis and cutaneous wound healing. Biomedicines 8, 101. 10.3390/biomedicines8050101 32365896 PMC7277690

[B7] Kant V. Jangir B. L. Sharma M. Kumar V. Joshi V. G. (2021). Topical application of quercetin improves wound repair and regeneration in diabetic rats. Immunopharmacol. Immunotoxicol. 43, 536–553. 10.1080/08923973.2021.1950758 34278923

[B8] Karar J. Maity A. (2011). PI3K/AKT/mTOR pathway in angiogenesis. Front. Mol. Neurosci. 4, 51. 10.3389/fnmol.2011.00051 22144946 PMC3228996

[B9] Li Z. Lin K. Wang Y. Mao J. Yin Y. Li Z. (2025). Garcinol promotes wound healing in diabetic mice by regulating inflammation and NLRP3 inflammasome-mediated pyroptosis via the PI3K/Akt/NF-κB pathway. Int. Immunopharmacol. 151, 114352. 10.1016/j.intimp.2025.114352 40022821

[B10] Lin Z.-Q. Kondo T. Ishida Y. Takayasu T. Mukaida N. (2003). Essential involvement of IL-6 in the skin wound-healing process as evidenced by delayed wound healing in IL-6-deficient mice. J. Leukoc. Biol. 73, 713–721. 10.1189/jlb.0802397 12773503

[B11] Louiselle A. E. Niemiec S. M. Zgheib C. Liechty K. W. (2021). Macrophage polarization and diabetic wound healing. Transl. Res. 236, 109–116. 10.1016/j.trsl.2021.05.006 34089902

[B12] Lv D. Cao X. Zhong L. Dong Y. Xu Z. Rong Y. (2023). Targeting phenylpyruvate restrains excessive NLRP3 inflammasome activation and pathological inflammation in diabetic wound healing. Cell Rep. Med. 4, 101129. 10.1016/j.xcrm.2023.101129 37480849 PMC10439185

[B13] Martin P. Leibovich S. J. (2005). Inflammatory cells during wound repair: the good, the bad and the ugly. Trends Cell Biol. 15, 599–607. 10.1016/j.tcb.2005.09.002 16202600

[B14] McDermott K. Fang M. Boulton A. J. M. Selvin E. Hicks C. W. (2023). Etiology, epidemiology, and disparities in the burden of diabetic foot ulcers. Diabetes Care 46, 209–221. 10.2337/dci22-0043 36548709 PMC9797649

[B15] Sharifiaghdam M. Shaabani E. Faridi-Majidi R. De Smedt S. C. Braeckmans K. Fraire J. C. (2022). Macrophages as a therapeutic target to promote diabetic wound healing. Mol. Ther. 30, 2891–2908. 10.1016/j.ymthe.2022.07.016 35918892 PMC9482022

[B16] Van Loo G. Bertrand M. J. M. (2023). Death by TNF: a road to inflammation. Nat. Rev. Immunol. 23, 289–303. 10.1038/s41577-022-00792-3 36380021 PMC9665039

[B17] Vergadi E. Ieronymaki E. Lyroni K. Vaporidi K. Tsatsanis C. (2017). Akt signaling pathway in macrophage activation and M1/M2 polarization. J. Immunol. 198, 1006–1014. 10.4049/jimmunol.1601515 28115590

[B18] Voza F. A. Huerta C. T. Le N. Shao H. Ribieras A. Ortiz Y. (2024). Fibroblasts in diabetic foot ulcers. Int. J. Mol. Sci. 25, 2172. 10.3390/ijms25042172 38396848 PMC10889208

[B19] Wang J. Hu K. Cai X. Yang B. He Q. Wang J. (2022). Targeting PI3K/AKT signaling for treatment of idiopathic pulmonary fibrosis. Acta Pharm. Sin. B 12, 18–32. 10.1016/j.apsb.2021.07.023 35127370 PMC8799876

[B20] Xue J. Ye B. Liu S. Cao S. Bian W. Yao C. (2020). Treatment efficacy of Chuang Ling Ye, a traditional Chinese herbal medicine compound, on idiopathic granulomatous mastitis: a randomized controlled trial. Evid-based Compl. Alt. 2020, 6964801. 10.1155/2020/6964801 32714413 PMC7341429

[B21] Yang D. Moh S. Son D. You S. Kinyua A. Ko C. (2016). Gallic acid promotes wound healing in normal and hyperglucidic conditions. Molecules 21, 899. 10.3390/molecules21070899 27399667 PMC6274221

[B22] Yang Y. Jia X. Qu M. Yang X. Fang Y. Ying X. (2023). Exploring the potential of treating chronic liver disease targeting the PI3K/Akt pathway and polarization mechanism of macrophages. Heliyon 9, e17116. 10.1016/j.heliyon.2023.e17116 37484431 PMC10361319

[B23] Yang J. Huang Z. Tan J. Pan J. Chen S. Wan W. (2024). Copper ion/gallic acid MOFs-laden adhesive pomelo peel sponge effectively treats biofilm-infected skin wounds and improves healing quality. Bioact. Mater. 32, 260–276. 10.1016/j.bioactmat.2023.10.005 37869725 PMC10589730

[B24] Yao Z. Xue K. Chen J. Zhang Y. Zhang G. Zheng Z. (2024). Biliverdin improved angiogenesis and suppressed apoptosis via PI3K/Akt-mediated Nrf2 antioxidant system to promote ischemic flap survival. Free Radic. Biol. Med. 225, 35–52. 10.1016/j.freeradbiomed.2024.09.042 39332540

[B25] Zhang Z. Wang L. Li X. Miao Y. Li D. (2024). Integrating network pharmacology, molecular docking and experimental validation to explore the pharmacological mechanisms of quercetin against diabetic wound. Int. J. Med. Sci. 21, 2837–2850. 10.7150/ijms.100468 39512686 PMC11539386

[B26] Zubair M. Ahmad J. (2019). Role of growth factors and cytokines in diabetic foot ulcer healing: a detailed review. Rev. Endocr. Metab. Disord. 20, 207–217. 10.1007/s11154-019-09492-1 30937614

