# Supplementary material for: Treatment of diabetic ulcers with Chuang Ling Ye: systems pharmacology and functional validation reveal the mechanism of angiogenesis and inflammation abatement driven by the PI3K-AKT signaling pathway
Source: Front Pharmacol. 2025 Oct 27;16:1644541. doi: 10.3389/fphar.2025.1644541 (PMC12597994; doi:10.3389/fphar.2025.1644541)
Supplement: Supplementary file 1 [file DataSheet1.pdf]

Supplementary data

No.	Content	Page
1	**Table S1** Complete list of metabolites identified by UHPLC-MS/MS in CLY.	1-9
2	**Fig. S1.** CCK-8 assay to determine the effect of CLY on THP-1 cell proliferation.	10
3	**Fig. S2.** Raw data of Cell scratch assay for Fig. 5B (n=3).	11
4	**Fig. S3.** Raw data of western blots for Fig. 3C (n=6).	12
5	**Fig. S4.** Raw data of western blots for Fig. 6A (n=3).	13
6	**Fig. S5.** Information of CLY.	14
7	**Fig. S6.** Expanded view of Figure 4A.	15
8	**Fig. S7.** Expanded view of Figure 4B.	16
9	**Fig. S8.** Expanded view of Figure 4C.	17

Metabolites in CLY

Table S1 Complete list of metabolites identified by UHPLC-MS/MS in CLY.

No.	tR/min	Identified component	Formula	Adduct	m/z
1	4.355367	Gallic Acid	C7H6O5	[M-H]-	169.0127
2	2.279567	Isoleucine	C6H13NO2	[M+H]+	132.1018
3	1.046767	Citric Acid	C6H8O7	[M-H]-	191.0185
4	1.279617	Pyroglutamic Acid	C5H7NO3	[M+H]+	130.0499
5	0.9041	Choline	C5H14NO	[M]+	104.1072
6	2.75205	Tyrosine	C9H11NO3	[M+H]+	182.081
7	0.95975	Proline	C5H9NO2	[M+H]+	116.0708
8	5.395817	Phenylalanine	C9H11NO2	[M+H]+	166.0862
9	0.876267	Arginine	C6H14N4O2	[M+H]+	175.1189
10	0.945783	Betaine	C5H11NO2	[M+H]+	118.0865
11	0.963133	Malic Acid	C4H6O5	[M-H]-	133.0126
12	11.12915	Oleamide	C18H35NO	[M+NH4]+	282.279
13	8.723233	Emodin	C15H10O5	[M-H]-	269.0452
14	0.87955	Bergapten	C12H8O4	[M-H]-	215.0317
15	2.2518	Piperidine	C5H11N	[M+H]+	86.0969
16	5.395817	Phenylethanolamine	C8H11NO	[M+H-H2O]+	120.0808
17	5.907217	Gallotannin	C27H24O18	[M-H]-	635.0894
18	1.7373	Adenine	C5H5N5	[M+H]+	136.0574
19	7.020216	7-Methoxy-4-Methylcoumarin	C11H10O3	[M+H]+	191.0703
20	0.893483	Glucose	C6H12O6	[M-H]-	179.0545
21	3.37775	2-Pyrrolidone	C4H7NO	[M+H]+	86.06053
22	1.265767	Valine	C5H11NO2	[M+H]+	118.0865
23	0.991133	Malonic Acid	C3H4O4	[M-H]-	103.0019
24	6.315017	Ellagic Acid	C14H6O8	[M-H]-	300.9977
25	7.202267	Gastrodin	C13H18O7	[M+Na]+	309.0867
26	1.046767	Galactose	C6H12O6	[M-H]-	179.0545
27	1.418317	Pipecolic Acid	C6H11NO2	[M+H]+	130.0861
28	1.3959	Shikimic Acid	C7H10O5	[M-H]-	173.044
29	7.200583	Endocrocin	C16H10O7	[M-H]-	313.0345
30	0.87955	Arabinonic Acid	C5H10O6	[M-H]-	165.0388
31	4.355367	Methyl 2-Furoate	C6H6O3	[M-H]-	125.0228
32	0.95975	Trigonelline	C7H7NO2	[M+H]+	138.055
33	0.87955	Gluconic Acid	C6H12O7	[M-H]-	195.0498
34	6.907467	Decursinol	C14H14O4	[M+H]+	247.0961
35	6.233783	Isoquercitrin	C21H20O12	[M+H]+	465.1033
36	2.903433	1,2,4-Benzenetriol	C6H6O3	[M-H]-	125.0228
37	5.368017	Adenosine	C10H13N5O4	[M+H]+	268.1032
38	0.987617	D-Pipecolic Acid	C6H11NO2	[M+H]+	130.0862
39	1.0155	Beta-D-Glucose	C6H12O6	[M+NH4]+	198.0972
40	8.04915	Rhein	C15H8O6	[M-H]-	283.0245
41	1.584617	Gamma-Guanidinobutyric Acid	C5H11N3O2	[M+H]+	146.0921
42	2.821517	2,3-Dihydrobenzofuran	C8H8O	[M+H]+	121.0649
43	5.898167	Tetrahydroharman-3-Carboxylic Acid	C13H14N2O2	[M+H]+	231.1124
44	6.91955	Genistin	C21H20O10	[M-H]-	431.0968

45	7.088167	Quercetin	C15H10O7	[M-H]-	301.0348
46	6.907467	Tinnevellin Glucoside	C20H24O9	[M+H]+	409.1494
47	5.521283	Pyrogallol	C6H6O3	[M+H]+	127.0391
48	6.3176	Liquiritin	C21H22O9	[M+H]+	419.134
49	1.6124	Cytidine	C9H13N3O5	[M+H]+	244.0922
50	2.0572	Nicotinamide	C6H6N2O	[M+H]+	123.0555
51	2.289767	Succinic Acid	C4H6O4	[M-H]-	117.0175
52	7.10415	Morin	C15H10O7	[M+H]+	303.0501
53	7.453383	Kaempferol	C15H10O6	[M-H]-	285.0392
54	1.667883	Nicotinic Acid	C6H5NO2	[M+H]+	124.0395
55	3.002133	Uridine	C9H12N2O6	[M-H]-	243.0618
56	3.002167	Salsolinol	C10H13NO2	[M+H]+	180.102
57	13.68077	4-Hydroxyacetophenone	C8H8O2	[M-H]-	135.0434
58	5.800267	D-Pyroglutamic Acid Ethyl Ester	C7H11NO3	[M+H]+	158.0809
59	5.702483	Maltol	C6H6O3	[M+H]+	127.0391
60	6.41545	Perlolyrine	C16H12N2O2	[M+H]+	265.0969
61	1.043267	Protoanemonin	C5H4O2	[M+H]+	97.02889
62	6.738733	Gossypetin	C15H10O8	[M+H]+	319.0445
63	2.821517	Tyramine	C8H11NO	[M+H]+	138.0912
64	6.3176	Cinnamic Acid	C9H8O2	[M-H2O+H]+	131.049
65	2.417067	Uric Acid	C5H4N4O3	[M-H]-	167.0195
66	6.440917	P-Coumaric Acid	C9H8O3	[M-H]-	163.0383
67	8.05885	Eudesmin	C22H26O6	[M+H]+	387.1799
68	3.016117	Uracil	C4H4N2O2	[M+H]+	113.0348
69	6.879517	2-Methoxycinnamaldehyde	C10H10O2	[M+H]+	163.075
70	1.626367	Cytosine	C4H5N3O	[M+H]+	112.0508
71	1.543067	Dimethyl Sulfoxide	C2H6OS	[M+H]+	79.02186
72	5.604867	Tryptophan	C11H12N2O2	[M+H]+	205.097
73	1.0294	Mannose	C6H12O6	[M+H-H2O]+	163.0598
74	6.328933	Benzoic Acid	C7H6O2	[M-H]-	121.0277
75	2.75205	4-Hydroxybenzaldehyde	C7H6O2	[M+H]+	123.0442
76	3.113633	Alloisoleucine	C6H13NO2	[M+H]+	132.1018
77	0.907367	Sorbose	C6H12O6	[M-H2O-H]-	161.0439
78	8.48525	Niranthin	C24H32O7	[M-H2O+H]+	415.2105
79	11.04242	Hexadecanamide	C16H33NO	[M+H]+	256.2629
80	5.381917	Guanosine	C10H13N5O5	[M+H]+	284.0982
81	0.963133	Maleic Acid	C4H4O4	[M-H]-	115.0018
82	7.200583	Flazine	C17H12N2O4	[M-H]-	307.0723
83	5.569467	Ethyl Benzoate	C8H8O4	[M-H]-	167.0333
84	1.778917	4-Aminophenol	C6H7NO	[M+H]+	110.0603
85	5.8143	1,2,3,4-Tetrahydro-Beta-Carboline-3-Carboxylic Acid	C12H12N2O2	[M+H]+	217.0972
86	2.015517	4-Nitrophenol	C6H5NO3	[M+H]+	140.034
87	8.399883	3,3',4',5,6,7,8-Heptamethoxyflavon	C22H24O9	[M+H]+	433.1498

		e			
88	6.989817	Rubiadin	C15H10O4	[M-H]-	253.0496
89	6.652033	Azelaic Acid	C9H16O4	[M-H]-	187.0962
90	0.84825	Propyl Gallate	C10H12O5	[M+H]+	213.0748
91	6.093717	Panasenoside	C27H30O16	[M+H]+	611.1605
92	6.342916	Ethyl Gallate	C9H10O5	[M-H]-	197.0441
93	6.91955	Salicylic Acid	C7H6O3	[M-H]-	137.0228
94	5.646783	Kojic Acid	C6H6O4	[M+H]+	143.034
95	0.862317	Theophylline	C7H8N4O2	[M+Na]+	203.0524
96	2.710317	L-Saccharopine	C11H20N2O6	[M+H-H2O]+	259.1289
97	6.17405	1,2,3,6-Tetragalloylglucose	C34H28O22	[M-H]-	787.0994
98	0.907367	Glycerate	C3H6O4	[M-H]-	105.0176
99	1.404483	Butyric Acid	C4H8O2	[M+Na]+	111.0444
100	0.9772	Trans-Aconitic Acid	C6H6O6	[M-H]-	173.0078
101	2.9453	Citraconic Acid	C5H6O4	[M-H]-	129.0176
102	7.2287	Sissotrine	C22H22O10	[M+FA-H]-	491.119
103	0.907367	Gulonolactone	C6H10O6	[M-H]-	177.039
104	6.567584	Spiraeoside	C21H20O12	[M-H]-	463.0867
105	0.893483	Glucuronic Acid	C6H10O7	[M-H]-	193.0343
106	6.024034	Quercetagitrin	C21H20O13	[M+H]+	481.0981
107	6.117533	Leucinic Acid	C6H12O3	[M-H]-	131.0696
108	7.23035	Acacetin	C16H12O5	[M+H]+	285.0751
109	8.25665	Nobiletin	C21H22O8	[M+H]+	403.1374
110	9.4247	Pc(16:0/22:6)	C46H80NO8P	[M+H]+	806.5653
111	1.917967	Pyridoxal	C8H9NO3	[M+H]+	168.0651
112	4.096633	Quinic Acid	C7H12O6	[M-H]-	191.0547
113	2.738083	Benzofuran	C8H6O	[M+H]+	119.0492
114	3.1579	Xanthine	C5H4N4O2	[M-H]-	151.0246
115	14.30213	2-Furanmethanol	C5H6O2	[M+H]+	99.04442
116	5.57705	1,6-Di-O-Galloylglucose	C20H20O14	[M+H]+	485.0914
117	5.457233	2,3-Dihydroxybenzoate	C7H6O4	[M-H]-	153.0208
118	6.469	Petunidin 3-O-Glucoside	C22H23O12+	[M-2H]-	477.1039
119	6.342916	Allose	C6H12O6	[M-H2O-H]-	161.0439
120	0.851717	Asparagine	C4H8N2O3	[M-H]-	131.0443
121	6.710433	Loliolide	C11H16O3	[M+H]+	197.1172
122	6.623867	Kaempferol 3-Rhamnoside	C21H20O10	[M-H]-	431.0969
123	6.807267	Zearalenone	C18H22O5	[M-H]-	317.1423
124	1.446067	Argininosuccinic Acid	C10H18N4O6	[M+H]+	291.1293
125	0.862317	Trimethyllysine	C9H20N2O2	[M+H]+	189.1621
126	4.25425	Cephalotaxine	C18H21NO4	[M+H]+	316.15
127	0.93525	Cellobiose	C12H22O11	[M+Cl]-	377.0851
128	11.30888	Erucamide	C22H43NO	[M+H]+	338.3419
129	5.654067	3,4-Dihydroxybenzoate	C7H6O4	[M-H]-	153.0178
130	6.117533	Rutin	C27H30O16	[M-H]-	609.1433
131	10.62353	6,10,14-Trimethyl-5,9,13-Pentadecatrien-2-	C18H30O	[M+H]+	263.237

		One			
132	6.244566	Gossypetin 8-Glucoside	C21H20O13	[M-H]-	479.0816
133	2.738083	Phenol	C6H6O	[M+H]+	95.04964
134	0.9041	Homoarginine	C7H16N4O2	[M+H]+	189.1344
135	6.107683	Cis-Anethole	C10H12O	[2M+H]+	149.0961
136	0.87955	D-Glutamic Acid	C5H9NO4	[M-H]-	146.0443
137	1.09895	1H-Pyrrole-2-Carboxaldehyde	C5H5NO	[M+H]+	96.0448
138	1.043267	Baikiain	C6H9NO2	[M+Na]+	128.0706
139	6.97805	Feruloyltyramine	C18H19NO4	[M+H]+	314.1375
140	0.931833	Turanose	C12H22O11	[M+NH4]+	360.1499
141	0.890167	Diacetyl	C4H6O2	[M+H]+	87.04452
142	0.93525	Sedoheptulose	C7H14O7	[M-H]-	209.0656
143	6.3176	Trans-Cinnamoyl Beta-D-Glucoside	C15H18O7	[M+H]+	311.1117
144	8.28625	Retusine	C19H18O7	[M+H]+	359.1116
145	6.047467	Myricetin-3-O-Galactoside	C21H20O13	[M-H]-	479.0817
146	5.8842	Sorbic Acid	C6H8O2	[M+H]+	113.0599
147	7.10415	Calycosin	C16H12O5	[M+H]+	285.0749
148	6.443517	Isorhamnetin 3-Glucoside	C22H22O12	[M+H]+	479.118
149	5.57705	Catechin	C15H14O6	[M+H]+	291.0855
150	5.381917	Guanine	C5H5N5O	[M+H]+	152.0565
151	1.200717	Oxalosuccinic Acid	C6H6O7	[M-H2O-H]-	189.0026
152	7.864267	Tricin	C17H14O7	[M-H]-	329.0654
153	5.688483	Sinapic Alcohol	C11H14O4	[M+H-H2O]+	193.0861
154	6.275683	Narcissin	C28H32O16	[M+H]+	625.1747
155	13.40293	Methenamine	C6H12N4	[M+H]+	141.1134
156	0.84825	Beta-Alanine	C3H7NO2	[M+H]+	90.05535
157	0.893483	Malonic Semialdehyde	C3H4O3	[M-H]-	87.00681
158	6.1637	4-Methyl-2,6-Dimethoxyphenol	C9H12O3	[M+H]+	169.0858
159	0.921333	D-Ribose	C5H10O5	[M-H]-	149.0438
160	0.851717	Histidine	C6H9N3O2	[M-H]-	154.0606
161	6.345617	Miscanthoside	C21H22O11	[M+H]+	451.1236
162	7.370567	Fa 18:3+1O	C18H30O3	[M+H]+	295.227
163	8.05885	Icariside F2	C18H26O10	[M+Na]+	425.1361
164	9.448334	Triptophenolide	C20H24O3	[M-H]-	311.1676
165	8.564917	Glycitein	C16H12O5	[M-H]-	283.0606
166	0.890167	GABA	C4H9NO2	[M+H-H2O]+	86.06053
167	0.973733	Stachydrine	C7H13NO2	[M+H]+	144.1018
168	5.604867	Geniposidic Acid	C16H22O10	[M+Na]+	397.1124
169	0.851717	Threonine	C4H9NO3	[M-H]-	118.0492
170	1.046767	Acetoacetaldehyde	C4H6O2	[M-H2O-H]-	85.02764
171	7.665517	Zingerone	C11H14O3	[M+Na]+	217.0859
172	6.68225	Madecassic Acid	C30H48O6	[M+H-H2O]+	487.3407
173	6.992033	Daidzein	C15H10O4	[M+H]+	255.0648
174	0.9041	Methionine Sulfoxide	C5H11NO3S	[M+H]+	166.0532
175	5.8143	Quercetin 3,4'-Diglucoside	C27H30O17	[M+H]+	627.1547

176	2.9313	Leucine	C6H13NO2	[M-H]-	130.0857
177	9.856717	Hydroquinidine	C20H26N2O2	[M-H]-	325.1837
178	6.342916	P-Coumaraldehyde	C9H8O2	[M-H]-	147.0433
179	0.87955	Aspartate	C4H7NO4	[M-H]-	132.0284
180	6.49725	Isookaninglucoside	C21H22O11	[M-H]-	449.1068
181	1.0571	Mannitol	C6H14O6	[M+H]+	183.086
182	7.635383	Arjugenin	C30H48O6	[M-H]-	503.3369
183	3.9486	Octopamine	C8H11NO2	[M+H]+	154.0861
184	9.4247	Pc(18:2/18:2)	C44H80NO8P	[M+H]+	782.5657
185	6.936117	3',4',7-Trihydroxyflavone	C15H10O5	[M+H]+	271.0598
186	9.4247	Sm(D18:2/16:0)	C39H77N2O6P	[M+CH3OH+H]+	701.5594
187	0.75035	Lysine	C6H14N2O2	[M+H]+	147.1129
188	6.907467	Ononin	C22H22O9	[M+H]+	431.1308
189	6.865334	Curcumol	C15H24O2	[M+H]+	237.1848
190	8.8993	5,7-Dimethoxyflavone	C17H14O4	[M+H]+	283.0959
191	7.00615	Berberine	C20H18NO4+	[M]+	336.1227
192	1.432217	Mannosamine	C6H13NO5	[M-H2O+H]+	162.0759
193	2.446383	Pyridoxine	C8H11NO3	[M+H]+	170.0808
194	6.289667	Sphondin	C12H8O4	[M+H]+	217.0495
195	0.681067	Spermidine	C7H19N3	[M+H]+	146.165
196	9.4247	Pc(18:1/14:0)	C40H78NO8P	[M+H]+	732.554
197	3.391567	2-Methylbutylamine	C5H13N	[M+H]+	88.11252
198	6.38495	Tropic Acid	C9H10O3	[M-H]-	165.0543
199	9.15525	Samidin	C21H22O7	[M+Na]+	409.1257
200	7.9351	Citreorosein	C15H10O6	[M-H]-	285.0391
201	7.13215	O-Methylsinapic Acid	C12H14O5	[M+H]+	239.0913
202	1.25185	Theanine	C7H14N2O3	[M+H]+	175.1075
203	8.104967	Physcione	C16H12O5	[M-H]-	283.0617
204	0.87955	Alanine	C3H7NO2	[M-H]-	88.03865
205	5.5073	Norvaline	C5H11NO2	[M-H2O+H]+	100.076
206	6.3176	Picrotin	C15H18O7	[M+Na]+	333.0945
207	7.87655	9,10-Anthracenedione, 1,3,7-Trihydroxy-2,8-Dimethoxy-6-Methyl-	C17H14O7	[M+H]+	331.0814
208	6.20235	Emodin 8-Glucoside	C21H20O10	[M-H]-	431.097
209	6.68225	Apigenin 7-Glucuronide	C21H18O11	[M+H]+	447.0909
210	5.758367	Chlorogenic Acid	C16H18O9	[M+H]+	355.1019
211	7.2164	Nandrolone	C18H26O2	[M+H]+	275.2005
212	5.499467	Glutaric Acid	C5H8O4	[M-H]-	131.0333
213	0.837867	Serine	C3H7NO3	[M-H]-	104.0336
214	2.50245	Gallic Acid Methyl Ester	C8H8O5	[M-H]-	183.0319
215	2.031717	3-Hydroxypicolinic Acid	C6H5NO3	[M-H]-	138.0178
216	5.758367	Kynurenic Acid	C10H7NO3	[M+H]+	190.0497
217	1.074583	Hydroxyacetic Acid	C2H4O3	[M-H]-	75.00547
218	5.8563	Roseoside	C19H30O8	[M+H]+	387.2016

219	5.800267	1-Naphthylamine	C10H9N	[M+H]+	144.0805
220	6.345617	Fraxidin	C11H10O5	[M+H]+	223.0596
221	6.7932	Aromadendrin	C15H12O6	[M-H]-	287.0557
222	5.521283	Acetylproline	C7H11NO3	[M+H]+	158.0809
223	5.954033	Luteolin-6-C-Glucoside	C21H20O11	[M+H]+	449.1061
224	7.836167	Cirsimaritin	C17H14O6	[M-H]-	313.0712
225	7.23035	Glycyrrhetinic Acid	C30H46O4	[M+H-H2O]+	453.3358
226	7.862333	Desacetyllaurenobiolide	C15H20O3	[M+NH4]+	249.1478
227	8.812567	Tectochrysin	C16H12O4	[M+H]+	269.0802
228	8.143267	Wogonin	C16H12O5	[M+H]+	285.0749
229	5.451617	2-Methyl-3-[(2S,3R,4S,5S,6R)-3,4,5-Trihydroxy-6-(Hydroxymethyl)Oxan-2-Yl]Oxypyran-4-One	C12H16O8	[M+H]+	289.0917
230	6.075517	Quercetin 3-Galactoside	C21H20O12	[M-H]-	463.0867
231	6.440917	2-Methylbenzaldehyde	C8H8O	[M-H]-	119.0485
232	6.933617	Phloretic Acid	C9H10O3	[M-H]-	165.0544
233	7.370567	Pinocarvyl Acetate	C12H18O2	[M+CH3OH+H]+	195.1381
234	6.583367	Scopoletin	C10H8O4	[M+H]+	193.0492
235	6.696434	Eupafolin	C16H12O7	[M+H]+	317.0649
236	7.735417	Formononetin	C16H12O4	[M-H]-	267.065
237	6.68225	Luteolin	C15H10O6	[M+H]+	287.0547
238	6.527534	Dopamine	C8H11NO2	[M+H-H2O]+	136.0758
239	6.287017	Kaempferol 3-O-Rutinoside	C27H30O15	[M-H]-	593.15
240	3.655117	GMP	C10H14N5O8P	[M-H]-	362.0493
241	8.0308	3',4',5',5,7-Pentamethoxyflavone	C20H20O7	[M+H]+	373.1272
242	8.148617	Pinocembrin	C15H12O4	[M-H]-	255.0651
243	6.950133	Palmatine	C21H22NO4+	[M]+	352.1537
244	6.936117	Paeonol	C9H10O3	[M+H]+	167.0701
245	6.131567	Glucobrassicin	C16H20N2O9S2	[M-H]-	447.0556
246	12.099	Stearic Acid Amide	C18H37NO	[M+H]+	284.2947
247	3.058433	Cis-Aconitate	C6H6O6	[M-H]-	173.0078
248	7.481467	3-Methylquercetin	C16H12O7	[M-H]-	315.0506
249	7.950117	3-O-Methylquercetin	C16H12O7	[M-H]-	315.0506
250	6.975717	Sebacic Acid	C10H18O4	[M-H]-	201.1117
251	6.287017	Anisole	C7H8O	[M-H]-	107.0485
252	5.5413	Benzoic Acid + 2O, O-Hex	C13H16O9	[M-H]-	315.0718
253	8.727317	Sm(D18:0/16:1)	C39H79N2O6P	[M+H]+	703.5731
254	8.522	Santin	C18H16O7	[M-H]-	343.0809
255	6.42945	4-Aminophenylacetic	C8H9NO2	[M+H]+	152.0704

		Acid			
256	6.261667	Astragalin	C21H20O11	[M+H]+	449.1082
257	11.12915	Octadeca-9,12-Dienal	C18H32O	[M+H]+	265.2525
258	8.356717	Darutoside	C26H44O8	[M+Na]+	507.2916
259	8.0351	1,3-Dihydroxyanthraquinone	C14H8O4	[M-H]-	239.034
260	5.674617	Gamma-Glutamylphenylalanine	C14H18N2O5	[M+H]+	295.1283
261	8.190516	6-Methylgingediol	C18H30O4	[M-H]-	309.2064
262	6.992033	Daidzin	C21H20O9	[M+Na]+	439.099
263	13.32675	Morpholine	C4H9NO	[M+H]+	88.07623
264	6.24775	Kaempferol 3-Glucuronide	C21H18O12	[M+H]+	463.086
265	9.4247	Pc(16:1/16:1)	C40H76NO8P	[M+H]+	730.5368
266	5.949317	Phthalic Acid	C8H6O4	[M-H]-	165.0177
267	6.009984	Myrtenal	C10H14O	[M+H]+	151.1118
268	5.395817	Guaiacol	C7H8O2	[M+H-H2O]+	107.0494
269	6.024034	Myricetin	C15H10O8	[M+H]+	319.0444
270	7.020216	Phebalosin	C15H14O4	[M+H]+	259.0963
271	6.3316	3-(Indol-3-Yl)Propionic Acid	C11H11NO2	[M+H]+	190.0859
272	5.78045	5-Caffeoylquinic Acid	C16H18O9	[M-H2O-H]-	353.0868
273	7.0179	Abscisic Acid	C15H20O4	[M-H]-	263.1281
274	5.995883	3-P-Coumaroylquinic Acid	C16H18O8	[M+H]+	339.1075
275	8.527534	6-Hydroxyluteolin 7,3',4'-Trimethyl Ether	C18H16O7	[M+CH3OH+H]+	345.0963
276	7.10415	Quinine	C20H24N2O2	[M+H]+	325.1975
277	9.754316	Pc(16:0/18:2)	C42H80NO8P	[M+H]+	758.5652
278	9.856717	Embelin	C17H26O4	[M-H]-	293.1783
279	5.381917	4-Hydroxybenzoylcholine	C12H18NO3	[M]+	224.128
280	6.595883	Pentagalloylglucose	C41H32O26	[M-H]-	939.1066
281	6.823383	Aloin	C21H22O9	[M+H]+	419.1339
282	8.48525	Beta-Methylstyrene	C9H10	[M+H-H2O]+	119.0858
283	5.479533	Pantothenic Acid	C9H17NO5	[M+H]+	220.1174
284	6.233783	P-Methoxycinnamic Acid Ethyl Ester	C12H14O3	[M+H]+	207.1014
285	6.303583	Visamminol	C15H16O5	[M+H]+	277.1064
286	6.485367	Vanillin	C8H8O3	[M+H]+	153.0546
287	8.05885	4-Methylbenzaldehyde	C8H8O	[M+H]+	121.0647
288	9.72585	Diisobutyl Phthalate	C16H22O4	[M+H]+	279.1588
289	7.23035	Curcumenol	C15H22O2	[M+H]+	235.1688
290	7.118134	O-Methylsinapic Acid	C12H14O5	[M+H-H2O]+	221.0807
291	9.624617	Morusin	C20H16O5	[M+H]+	337.107

		Hydroperoxide			
292	6.244566	4-Methoxysalicylic Acid	C8H8O4	[M-H]-	167.0334
293	7.10415	Jasmonic Acid	C12H18O3	[M+H]+	211.1328
294	6.794617	Xanthyletin	C14H12O3	[M+H]+	229.0856
295	6.287017	Suberic Acid	C8H14O4	[M-H]-	173.0802
296	7.51085	Chrysoeriol	C16H12O6	[M+H]+	301.0699
297	8.04915	Purpurin	C14H8O5	[M-H]-	255.0293
298	6.950133	Tiliroside	C30H26O13	[M+H]+	595.1436
299	13.48645	GlcNAc	C8H15NO6	[M+H-H2O]+	204.0863
300	5.912066	Coniferin	C16H22O8	[M+Na]+	365.1224
301	7.174317	Arjunic Acid	C30H48O5	[M-H2O+H]+	471.3463
302	8.214483	9-HOTrE	C18H30O3	[M+H-H2O]+	277.216
303	5.828283	2-Methylpropanoic Acid	C4H8O2	[M+Na]+	111.0444
304	7.94645	Ingenol	C20H28O5	[M+H]+	349.1984
305	7.79235	Isosinensetin	C20H20O7	[M+H]+	373.1273
306	5.5073	Metacetamol	C8H9NO2	[M+H]+	152.0708
307	7.23035	Glycitin	C22H22O10	[M+Na]+	469.1105
308	7.454484	Vitamin K1	C31H46O2	[M+2H]+	226.1802
309	6.24775	Aesculetin	C9H6O4	[M+H]+	179.0335
310	0.768133	Myo-Inositol 1-Phosphate	C6H13O9P	[M-H]-	259.0214
311	8.499367	Gardenin A	C21H22O9	[M+H]+	419.1339
312	7.118134	Geissoschizine Methyl Ether	C22H26N2O3	[M+H]+	367.209
313	8.003034	Arnicolide C	C19H26O5	[M+H]+	335.1828
314	6.922117	Prunin	C21H22O10	[M+H]+	435.128
315	6.41545	Catalposide	C22H26O12	[M+Na]+	505.1339
316	5.57705	Higenamine	C16H17NO3	[M+H]+	272.1273
317	5.744383	Caffeic Acid	C9H8O4	[M+H-H2O]+	163.0389
318	6.907467	Demethoxyyangonin	C14H12O3	[M+H]+	229.086
319	13.38155	1,2,3-Tri-O-Galloyl-Beta-D-Glucose	C27H24O18	[M-H]-	635.0892
320	8.290216	Corymbosin	C19H18O7	[M-H]-	357.0976
321	6.696434	Cinnamamide	C9H9NO	[M+H]+	148.0757
322	9.4247	Pc(15:0/15:0)	C38H76NO8P	[M+H]+	706.5382
323	7.202267	Indirubin	C16H10N2O2	[M+H]+	263.0797
324	5.429266	1,5-Bis(4-Hydroxy-3-Methoxyphenyl)-1,4-Pentadien-3-One	C19H18O5	[M-H]-	325.1071
325	5.828283	Riboflavin	C17H20N4O6	[M+H]+	377.1446
326	5.940067	Beta-Carboline	C11H8N2	[M+H]+	169.076
327	4.168583	Ciliatine	C2H8NO3P	[M-H]-	124.0146
328	6.093717	Xanthoxyline	C10H12O4	[M+H]+	197.0804
329	6.992033	Wogonoside	C22H20O11	[M+H]+	461.1073
330	8.555833	Tangeretin	C20H20O7	[M+Na]+	395.1104
331	7.96065	Apigenin Dimethylether	C17H14O5	[M+H]+	299.0915
332	6.177683	Rutarin	C20H24O10	[M+Na]+	447.127
333	7.7636	Caryophyllene Epoxide	C15H24O	[M+H]+	221.1898
334	6.794617	Piceid	C20H22O8	[M+H]+	391.139
335	8.073017	6-	C19H18O6	[M+H]+	343.1169

		Demethoxytangeretin			
336	8.541433	Coronaric Acid	C18H32O3	[M+H]+	297.2421
337	8.0308	5-Demethylnobiletin	C20H20O8	[M+H]+	389.1217
338	7.202267	Wedelolactone	C16H10O7	[M+H]+	315.0492
339	8.88195	9,10-Dihydroxystearic Acid	C18H36O4	[M-H]-	315.2533
340	9.72585	Phthalic Anhydride	C8H4O3	[M+H]+	149.0231
341	8.328433	4',5,6,7-Tetramethoxyflavone	C19H18O6	[M+H]+	343.1167
342	8.290216	7-Hydroxyflavone	C15H10O3	[M-H]-	237.0549
343	6.992033	Flindersine	C14H13NO2	[M+H]+	228.1014
344	8.04475	Inosine	C10H12N4O5	[M+H]+	269.0916
345	13.38905	Di-N-Propylamine	C6H15N	[M+H]+	102.1281
346	7.93245	N,N-Dimethyldodecylamine	C14H31N	[M+H]+	214.2525
347	9.26865	Dihydrosamidin	C21H24O7	[M+Na]+	411.1403
348	6.879517	Lapachol	C15H14O3	[M+H]+	243.1012
349	9.40425	Valerenic Acid	C15H22O2	[M-H]-	233.1539
350	9.185317	5-O-Methylgenistein	C16H12O5	[M-H]-	283.0606
351	10.91162	Decamethylcyclopentasiloxane	C10H30O5Si5	[M+H]+	371.1001
352	9.639283	Corynoxeine	C22H26N2O4	[M+H]+	383.2042
353	8.8993	6,7-Dimethoxyflavone	C17H14O4	[M+Na]+	305.0777
354	8.148617	Skullcapflavone II	C19H18O8	[M-H]-	373.0923
355	8.59305	Asiatic Acid	C30H48O5	[M+FA-H]-	533.3481
356	13.34257	Homocysteine	C4H9NO2S	[M+H]+	136.0479
357	11.42875	Kaempferol 3-Gentiobioside	C27H30O16	[M+Na]+	633.1481

Effect of CLY on THP-1 Cell Viability (CCK-8 Assay)

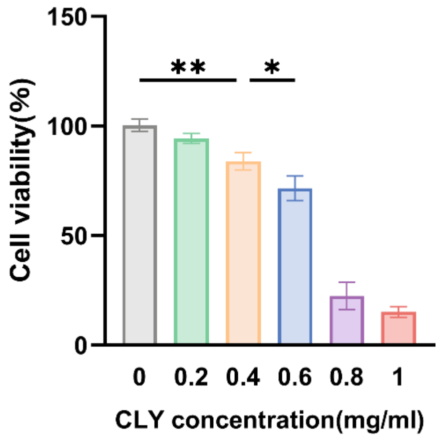

Fig. S1. CCK-8 assay to determine the effect of CLY on THP-1 cell proliferation.

Compared with the 0.4 mg/mL CLY group, * P≤0.05, ** P≤0.01

Raw data of Cell scratch assay

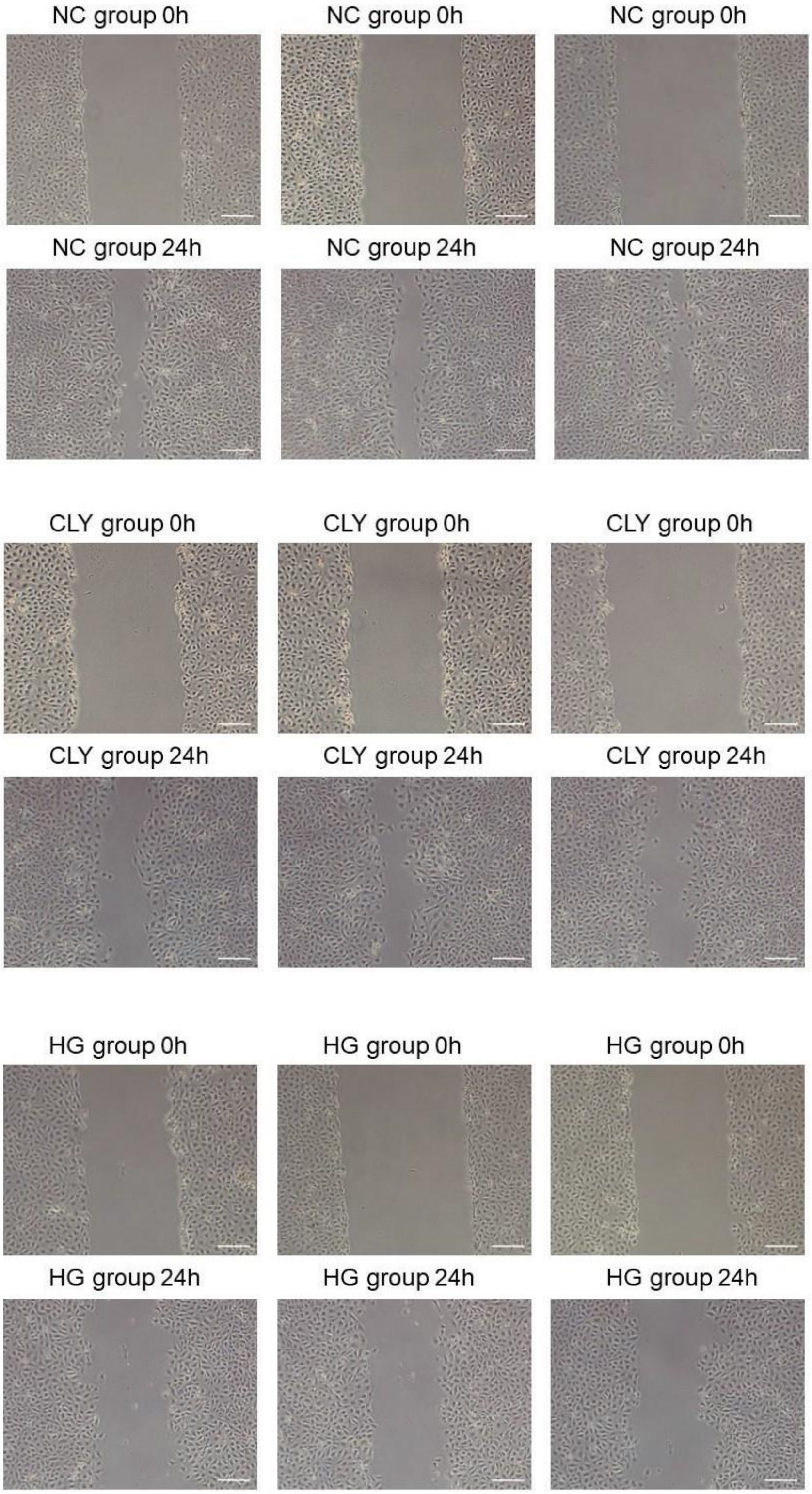

Fig. S2. Raw data of Cell scratch assay for Fig. 5B (n=3).

Raw data of western blots

Fig 3C

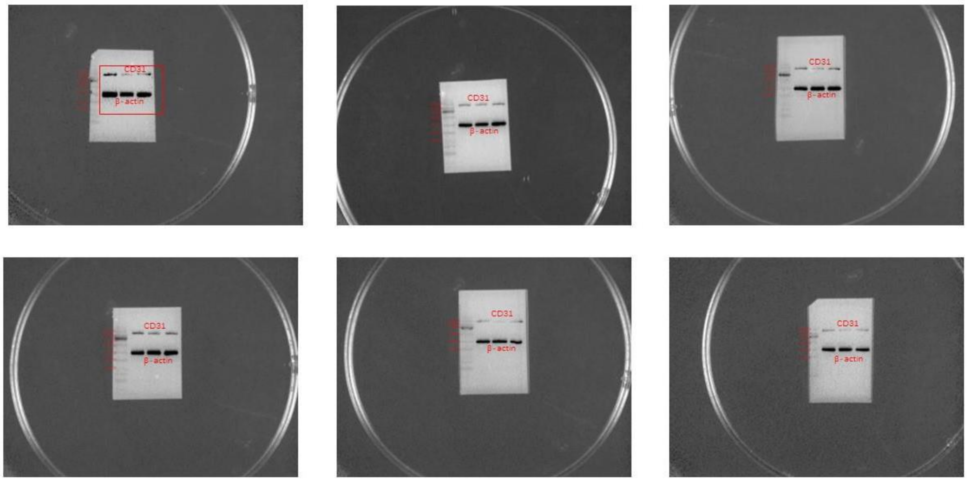

Fig 3C

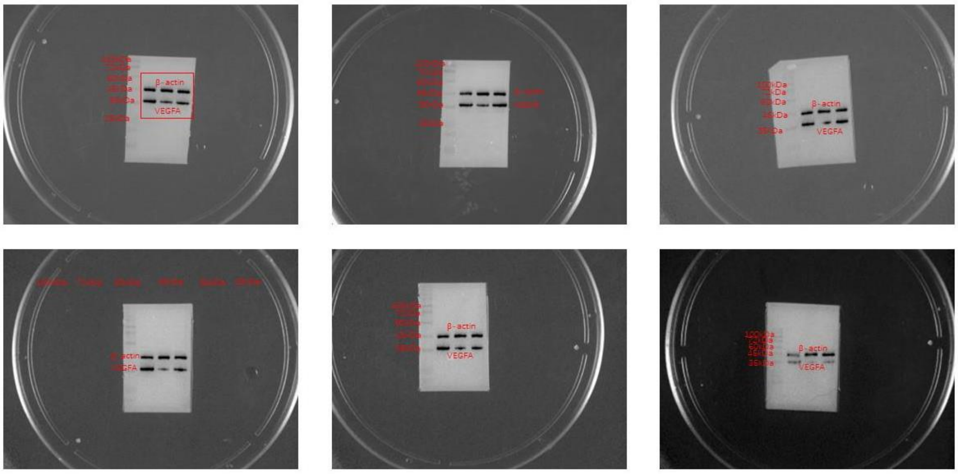

Fig. S3. Raw data of western blots for Fig. 3C (n=6).

Fig 6A

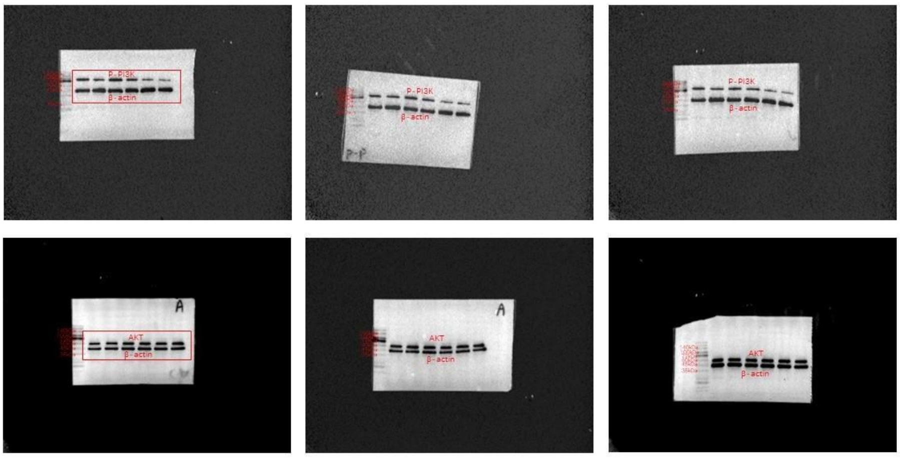

Fig 6A

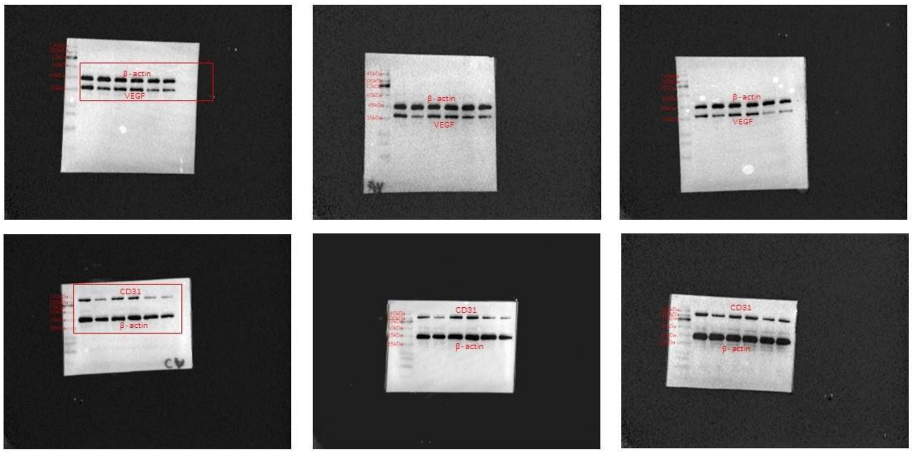

Fig 6A

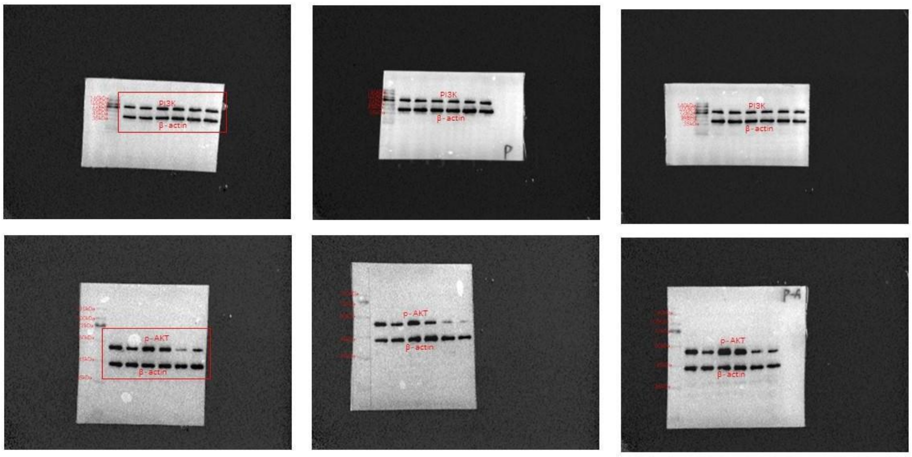

Fig. S4. Raw data of western blots for Fig. 6A (n=3).

Information of CLY

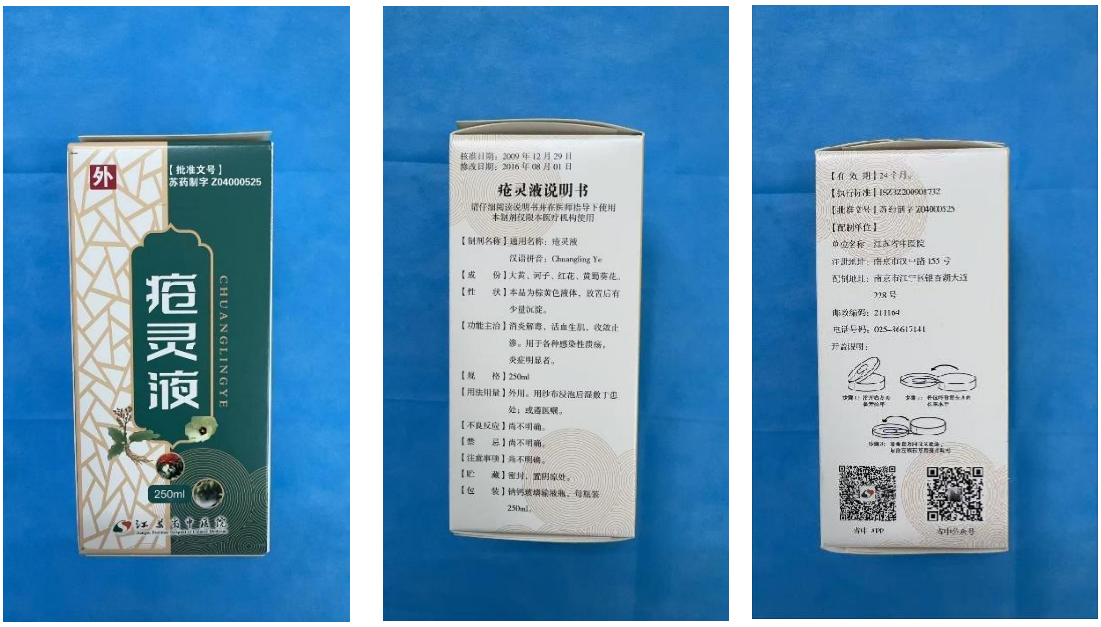

Fig. S5. Infourmation of CLY.

Expanded view of Figure 4

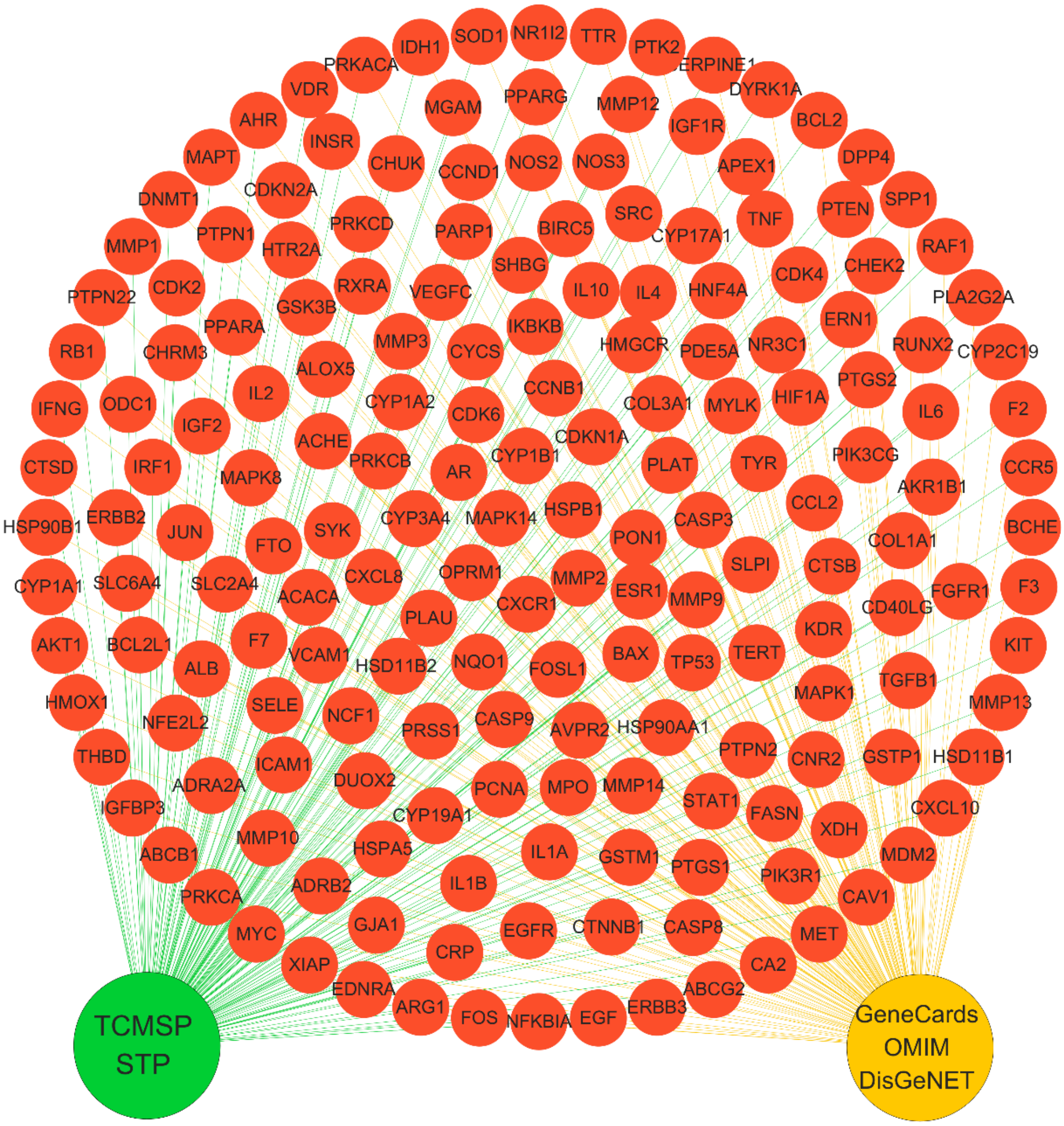

Fig. S6. Expanded view of Figure 4A.

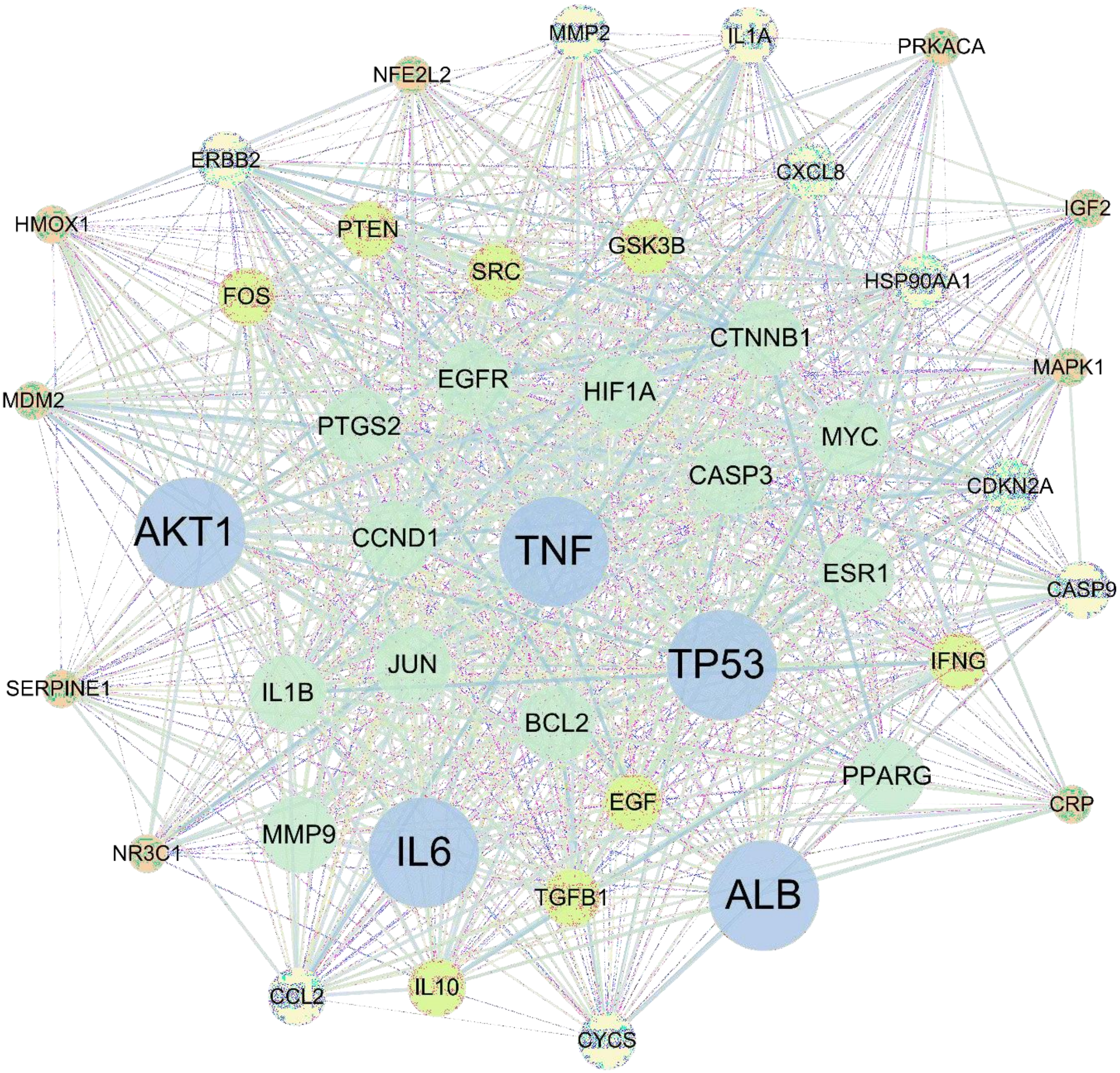

Fig. S7. Expanded view of Figure 4B.

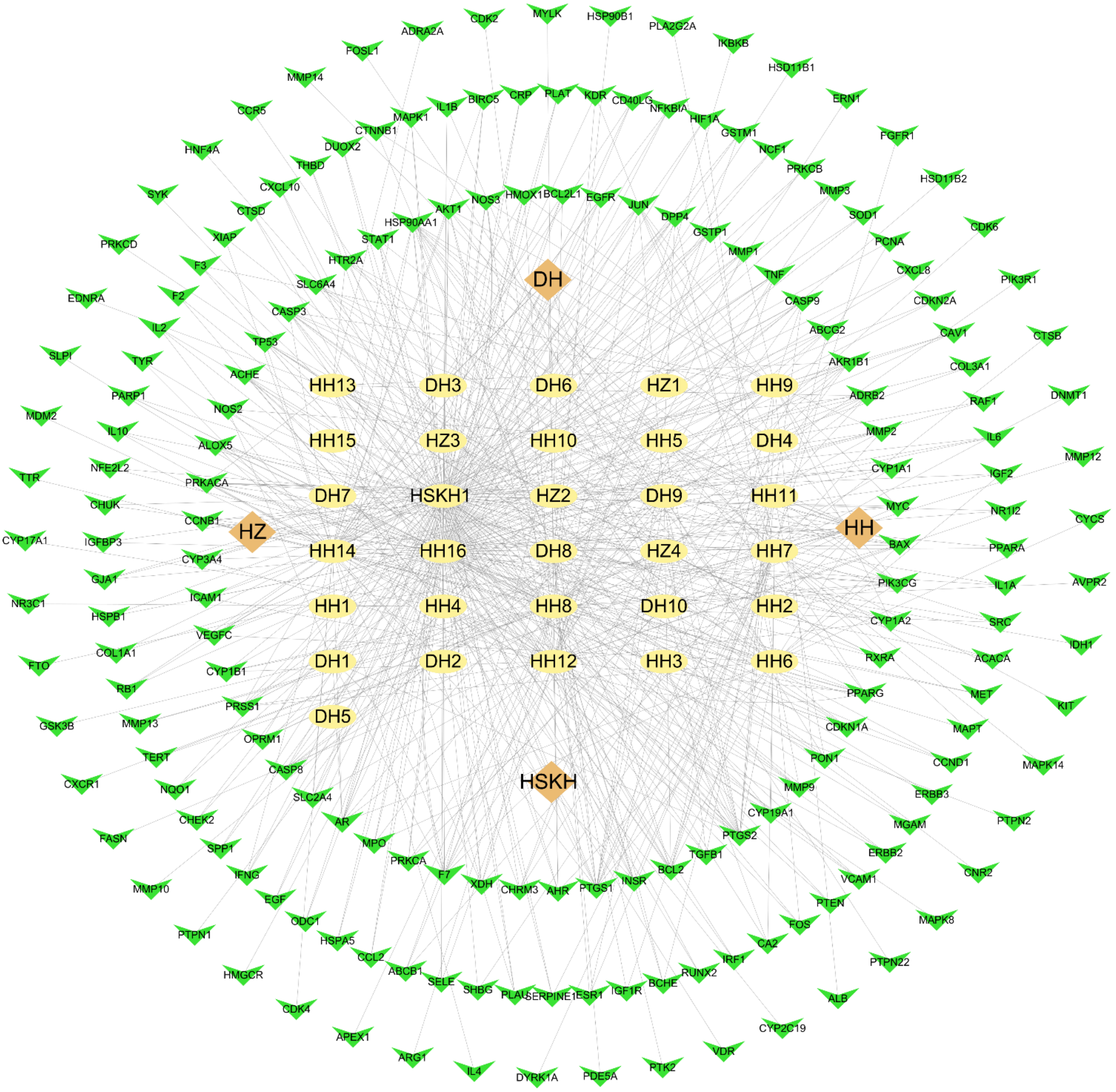

Fig. S8. Expanded view of Figure 4C.
